# Development of a Csy4-processed guide RNA delivery system with soybean-infecting virus ALSV for genome editing

**DOI:** 10.1186/s12870-021-03138-8

**Published:** 2021-09-13

**Authors:** Yanjie Luo, Ren Na, Julia S. Nowak, Yang Qiu, Qing Shi Lu, Chunyan Yang, Frédéric Marsolais, Lining Tian

**Affiliations:** 1 London Research and Development Centre, Agriculture and Agri-Food Canada, N5V 4T3 London, ON Canada; 2 Institute of Cereal and Oil Crops, Hebei Academy of Agricultural and Forestry Sciences, Shijiazhuang, 050031 China; 3 Institute of Vegetables and Flowers, Chinese Academy of Agricultural Sciences, Beijing, 100081 China

**Keywords:** CRISPR-Cas9, genome editing, virus vector, soybean, Golden Gate assembly

## Abstract

**Background:**

A key issue for implementation of CRISPR-Cas9 genome editing for plant trait improvement and gene function analysis is to efficiently deliver the components, including guide RNAs (gRNAs) and Cas9, into plants. Plant virus-based gRNA delivery strategy has proven to be an important tool for genome editing. However, its application in soybean which is an important crop has not been reported yet. ALSV (*apple latent spherical virus*) is highly infectious virus and could be explored for delivering elements for genome editing.

**Results:**

To develop a ALSV-based gRNA delivery system, the Cas9-based Csy4-processed ALSV Carry (CCAC) system was developed. In this system, we engineered the soybean-infecting ALSV to carry and deliver gRNA(s). The endoribonuclease Csy4 effectively releases gRNAs that function efficiently in Cas9-mediated genome editing. Genome editing of endogenous phytoene desaturase (*PDS*) loci and exogenous 5-enolpyruvylshikimate-3-phosphate synthase (*EPSPS*) sequence in *Nicotiana. benthamiana* (*N. benthamiana*) through CCAC was confirmed using Sanger sequencing. Furthermore, CCAC-induced mutagenesis in two soybean endogenous *GW2* paralogs was detected.

**Conclusions:**

With the aid of the CCAC system, the target-specific gRNA(s) can be easily manipulated and efficiently delivered into soybean plant cells by viral infection. This is the first virus-based gRNA delivery system for soybean for genome editing and can be used for gene function study and trait improvement.

**Supplementary Information:**

The online version contains supplementary material available at 10.1186/s12870-021-03138-8.

## Background

CRISPR/Cas9 (Clustered Regularly Interspaced Short Palindromic Repeats/CRISPR-associated 9) system is a powerful genome editing tool for plant gene function studies and crop trait improvement. The system originally evolved in the bacterial immune system and has been used for site-specific modifications in many plants including soybean (Cai et al. [5]; Cao et al. [6]; Chilcoat et al. [8]; Sun et al. [33]). Generally, it has two components: Cas9 nuclease and gRNA (guide RNA). To achieve targeted modification, these two components need to be delivered into plant cells. The targeting specificity of Cas9 is achieved by the 20 bp (bp) spacer sequence of gRNA (Jinek et al. [18]).

In most cases, the Cas9 and gRNA are cloned into a single or two separate binary vectors and subsequently transferred into plant cells usually mediated by *Agrobacterium* (Feng et al. [12]). One disadvantage of this method is that each binary construct is target-specific, and therefore if the target is changed a new construct and a new plant transformation are required. Whereas the transformation is usually time-consuming and labor-costing. Also, efficient transformation has not been developed for many plant species. These inhibit CRISPR/Cas9 based genome editing application in many crops, such as soybean.

Due to its delivery by virus infection instead of traditional transformation, the recently developed virus delivery gRNA system provides a promising approach to facilitate the application of CRISPR/Cas9 in plants (Ali et al. [2, 3]; Cody et al. [9]; Hu et al. [14]; Jiang et al. [17]; Yin et al. [43]). Some plant viruses can be developed into virus-based vectors for the delivery of exogenous nucleotides into plant cells for protein expression or virus-induced gene silencing (VIGS) (Zhang and Ghabrial [45]). Generally, the large size of foreign nucleotides can affect virus replication and movement, constraining the cargo-carrying capacity of the virus vectors (Thomas et al. [35]). Although it may be difficult that the plant virus vectors tolerate the insertion of a Cas9 ORF (around 4,100 bp), it is reported that they can carry and deliver the gRNA sequence (around 100 bp) into Cas9-expressing plant. Cas9 and gRNA can be introduced into plants using this approach via two independent methods: Cas9 is cloned into a binary vector and introduced to plant cells by traditional *Agrobacterium*-mediated transformation method, whereas gRNA is delivered into plants by virus infection. To date, several viruses have been reported to deliver gRNA through systemic infection in crops including wheat and maize using *barley stripe mosaic virus* (BSMV) (Hu et al. [14]), and sugar beet using *beet necrotic yellow vein virus* (BNYVV) (Jiang et al. [17]). However, each virus has a specific host range and variable infection efficiencies in different hosts. For a given plant species, it is essential to choose a specific highly infectious virus to develop a corresponding gRNA delivery system. This virus-induced delivery of gRNA has not been previously applied for the economically important soybean crop.

Although many viruses have been shown to infect soybean, ALSV (*apple latent spherical virus*) is highly infectious and has been previously used as vectors for inducing VIGS (viral-induced gene silencing) and expressing protein in soybean (Yamagishi and Yoshikawa [40, 41]). In this study, ALSV was studied to develop a gRNA delivery system, named CCAC (Cas9 based Csy4-processed ALSV Carry System). We tested the CCAC system in the following ways: (1) delivery of gRNA into *Nicotiana benthamiana* plants; (2) gRNA released from CCAC directing Cas9-mediated genome editing; (3) multiple targeting with CCAC in *N. benthamiana* and soybean. Our results indicated that CCAC functions efficiently in Cas9-mediated genome editing in plants. It provides an approach to facilitate the application of CRISPR/Cas9 in soybean by the target-specific gRNA(s) that can be easily programmed-and-reprogrammed and delivered into the plant for effective virus infection.

## Results

### Establishment of CCAC

**Fig. 1:**
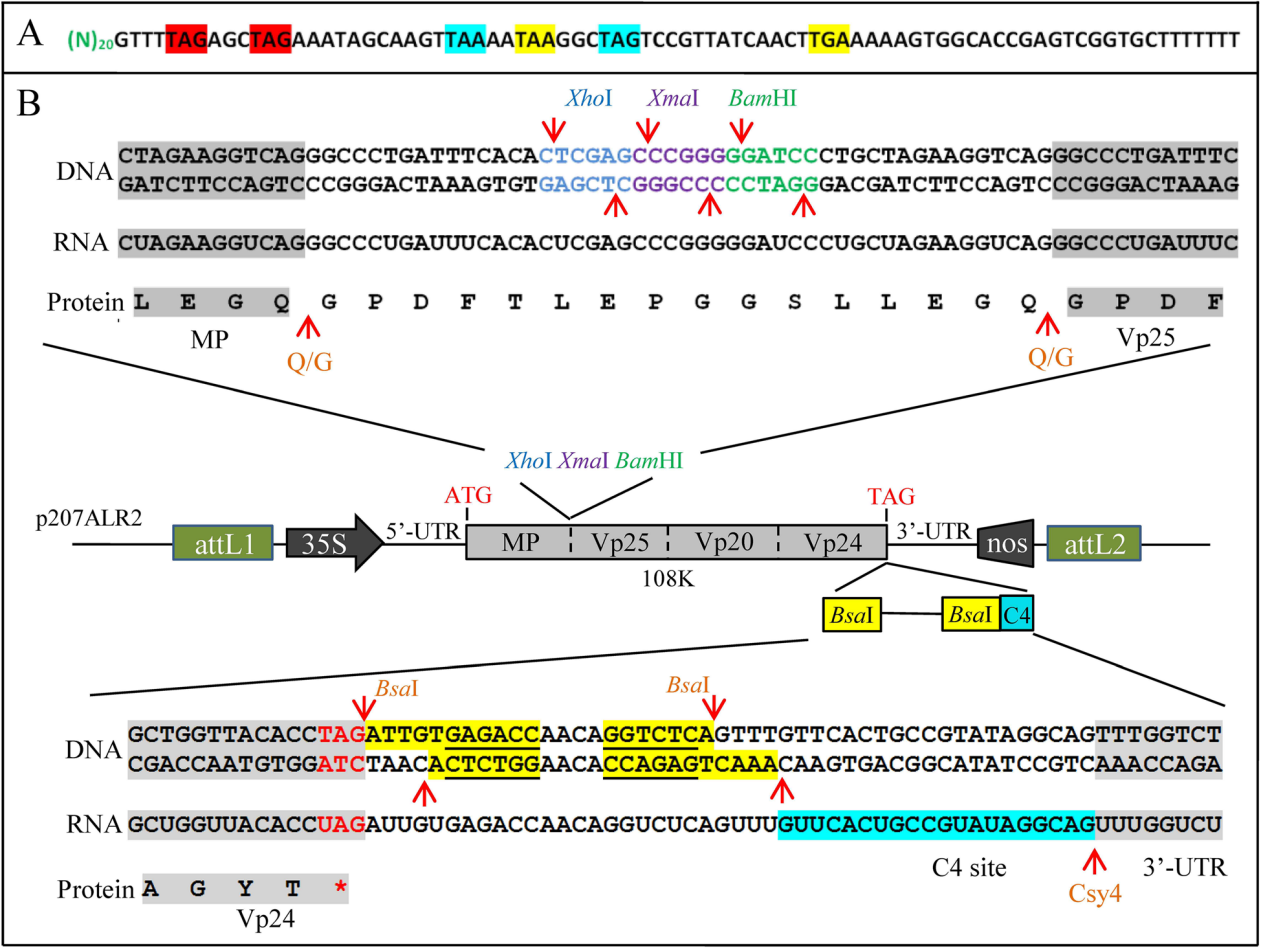
Development of CCAC. (**A**) Stop codon analysis of gRNA. (N)_20_denotes the 20 bp spacer sequence. Stop codons in frame 1 are highlighted in red, in frame 2 highlighted in yellow, and in frame 3 highlighted in blue. (**B**) Schematic representation of ALSV-RNA2 vector. The multiple cloning site (MCS)*Xho*I-*Xma*I-*Bam*HI is located between the artificial Q/G protease cleavage sites. The *Bsa*I sites were introduced immediately downstream of the translation stop codon of the Vp24 protein of 108k polyprotein, following the C4 site. attL1 and attL2 sequences are flanking the ALSV-RNA2 expression cassette. *Bsa*I sites are highlighted in yellow, while their recognition sites are also underlined. C4 site is highlighted in blue. The red arrows represent the various cleavage sites

To provide a virus-based gRNA delivery system for soybean, we used the soybean highly infecting virus ALSV to develop a gRNA delivery system, named as CCAC system. In the gRNA scaffold sequence, frame analysis illustrated that there are two stop codons in each of the three reading frames (Fig. 1 A). Since ALSV-RNA2 encodes a polyprotein in a single open reading frame, to avoid disrupting its translation, the downstream of the translation stop codon of the Vp24 protein in ALSV-RNA2 was chosen for insertion of gRNA in the CCAC system. The Csy4 recognition sequence (C4 site) was adopted for Csy4 to release mature gRNA from the virus genome (Fig. 1B).

**Fig. 2:**
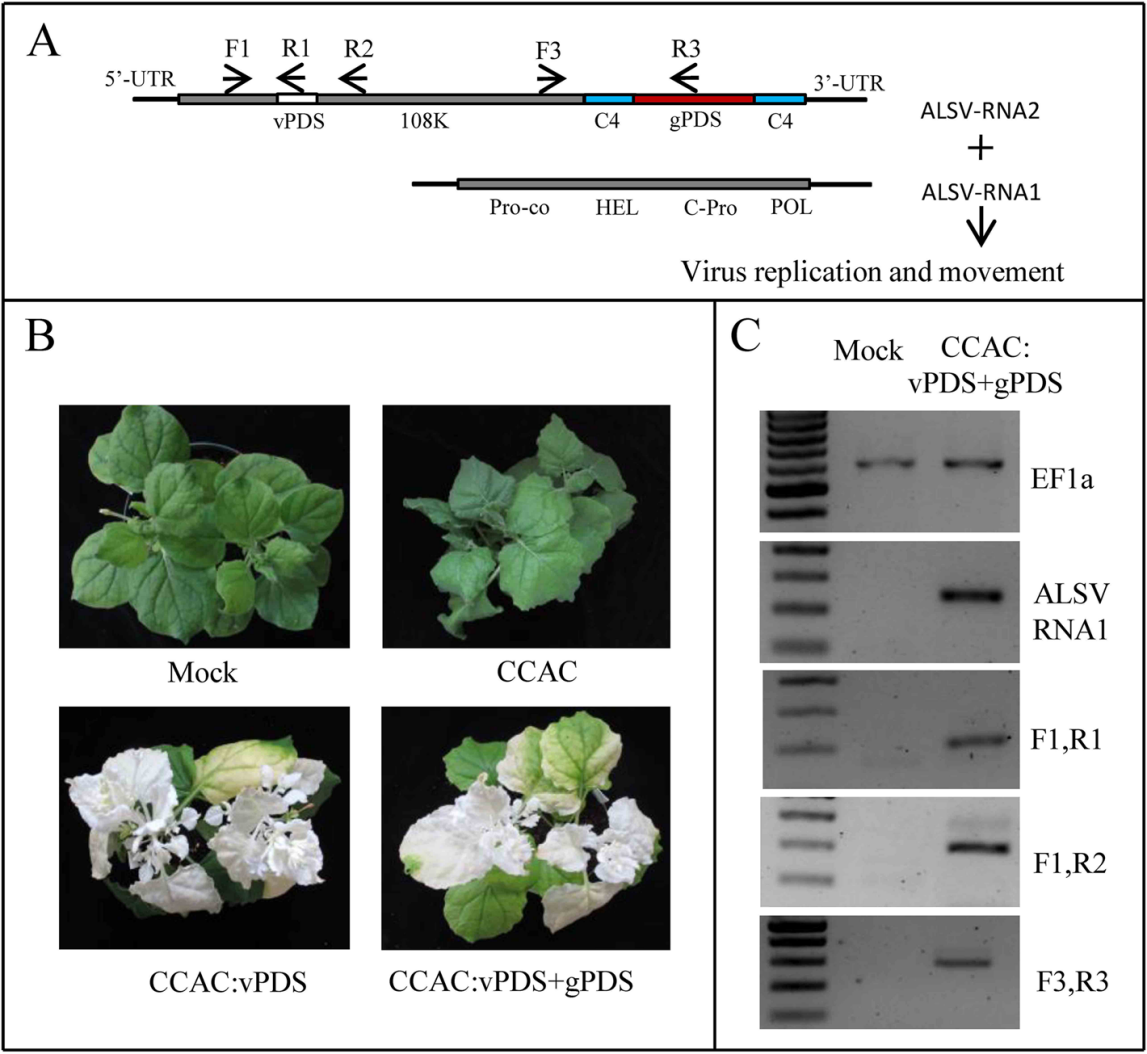
Infectivity of CCAC. (**A**) Diagram of CCAC:vPDS + gPDS. The gPDS (gRNA targeting PDS gene) flanked by two C4 sites, cloned downstream of 108k polyprotein. A 102 bp fragment of PDS (shown as “vPDS”) inserted into MCS, to silence PDS gene by VIGS. (**B**) Plants were agroinoculated with empty vector (Mock), p301ALR1 + p301ALR2(CCAC), p301ALR1 + p301ALR2-vPDS (CCAC:vPDS) or p301ALR1 + p301ALR2- vPDS -gPDS (CCAC:vPDS + gPDS). The photobleaching of leaves was observed in CCAC:vPDS and CCAC:vPDS + gPDS. Photos were taken 3 weeks post-inoculation. (**C**) Analysis of mRNAs in virus infected plants by RT-PCR. Total RNA was extracted from plants inoculated by Mock or CCAC:vPDS+gPDS (v+g). For “v+g”, the leaves of which one third of the surface area had been photobleached were used for RNA extraction. The housekeeping gene EF1a, and ALSV-RNA1 expression were used as the control. The positions of primers F1, R1, R2, F3 and R3 to detect ALSV-RNA2 were shown in A. Uncropped full-length gels of Figure 2C is shown in Supplementary Figure 8

Since the sequences in CCAC have been modified from natural ALSV viral RNAs, which may lead the virus to lose its biological activity, we first tested the ability of the plant to be infected by VIGS. Partial *PDS* (*phytoene desaturase*) gene sequence was inserted into the MCS site of CCAC (Fig. 2 A), then agroinfiltrated into *N. benthamiana* plants. The photobleaching phenotype was observed at the newly emerging leaves showing systemic infection by viral RNAs derived from the CCAC in the inoculated plants (Fig. 2B). This result indicated that CCAC can systemically infect plants and can be used for *Agrobacterium*-mediated virus inoculation. Next, we further investigated whether or not the gRNA, which is immediately integrated downstream of the Vp24 stop codon, will disrupt the biological activity of the virus. A *PDS*-targeting gRNA fragment flanked by two C4 sites was constructed into a *PDS* fragment (as a visible indicator caused by VIGS) containing CCAC, and then agroinfiltrated into *N. benthamiana* plants. The upper newly emerging leaves of the infiltrated plants were photobleached (Fig. 2B), demonstrating systemic plant infection. RT-PCR was performed to confirm the viral infection (Fig. 2 C, Additional file 2 Figure S1). These results indicated that CCAC can therefore be used to effectively carry and deliver gRNA with C4 sites.

### Cas9-induced genome editing with CCAC

**Fig. 3:**
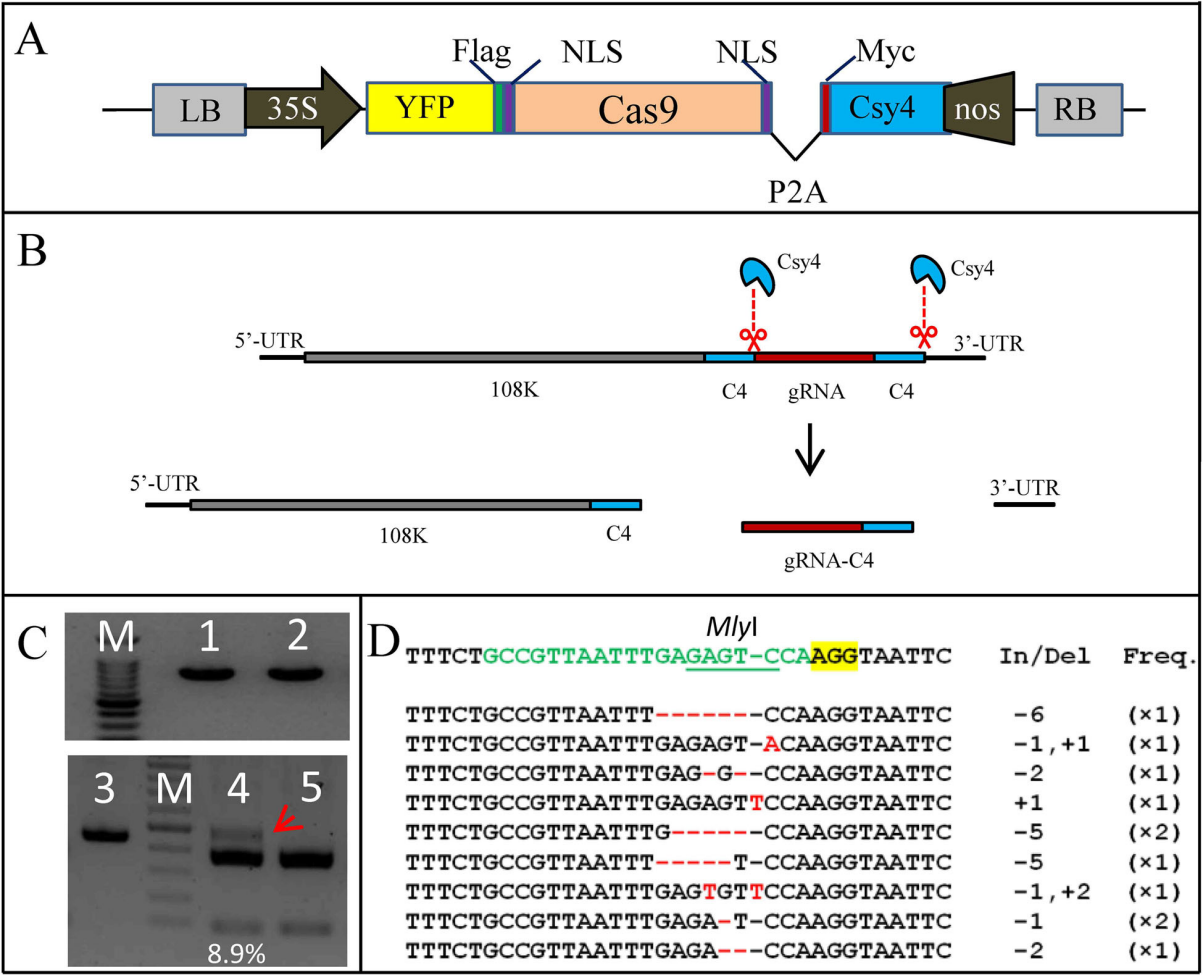
Cas9-induced genome editing with CCAC. (**A**) Schematic of Cas9-Cys4 construct. The YFP protein was fused to Cas9, while the Csy4 protein was separated by 2 A self-cleaving peptide (P2A). (**B**) gRNA release by Csy4 processing. The gRNA flanked by C4 sites, was replicated and moved along with ALSV virus RNAs. Csy4 cleaves and releases gRNA from the virus RNAs. The C4 site remains at the 3’ end of the gRNA. (**C**) Genome editing of PDS with CCAC. The photobeached leaves of “CCAC:vPDS+gPDS” plants in Figure 2B were or were not (as control) infiltrated with Cas9-Cys4 construct. 2dpi the genomic DNA was extracted and used as PCR template with primers flanking PDS locus. Lane 1 and lane 3 were the PCR products with photobleached leaves infiltrated by Cas9-Cys4 construct as template, while in lane 2 the template was the photobleached leaves without Cas9-Cys4 construct infiltration as control. Lanes4 and 5 show the MlyI -digested PCR products from lanes 1 and 2, respectively. The mutation rate (8.9%) was calculated by ImageJ. The red arrow indicates the MlyI -resistant band. M = DNA marker. Uncropped full-length gels of Figure 3C is shown in Supplementary Figure 9. (**D**) Alignment of PDS sequences with Cas9-induced indels obtained from the red arrow indicated band in C. The wild type sequence is shown at the top. The sequence targeted by gPDS is shown in green, whereas the mutations varying from that sequence are shown in red. PAM is highlighted in yellow. The total length (as indicated by number of bases) of indels (In/Del) and the frequency with which each DNA sequence pattern was observed (Freq.) are presented to the right of the sequences

We further tested the biological activity of gRNA carried by CCAC. We built a construct to co-express Cas9 and Csy4 (Cas9-Csy4 construct), in which the self-cleaving 2 A peptide (P2A) was used to separate Cas9 and Csy4 in one single ORF (Fig. 3 A, 3B). To investigate if the plant endogenous target was edited by Cas9, the upper newly emerging leaves of *N. benthamiana* plants systematically infected by CCAC carrying gPDS were agroinfiltrated with the Cas9-Csy4 construct. PCR-RE analysis results showed a portion of PCR amplicons (8.9 %) from plants infiltrated with the Cas9-Csy4 construct were *Mly*I-resistant (Fig. 3 C, Additional file 2 Figure S2), indicating that the *PDS* gene was edited by the Cas9 nuclease. Sanger sequencing results further confirmed the presence of indels (insertion-deletion polymorphisms) at the *PDS* gene target site (Fig. 3D, Additional file 3). In addition, 12 potential off-target sites of gPDS were tested using the restriction enzyme site loss method (Nekrasov et al. [28]). From the 12 loci, nine showed the expected PCR band (Additional file 1 Figure S1). None of the nine amplicons showed evidence of *Mly*I site loss as observed in the *PDS* target sequence. The results suggest that gRNA released by Csy4 nuclease from the ALSV virus genome in systemic leaves was biologically active during Cas9-mediated genome editing.

### Cas9-induced multiple targeting with CCAC

**Fig. 4:**
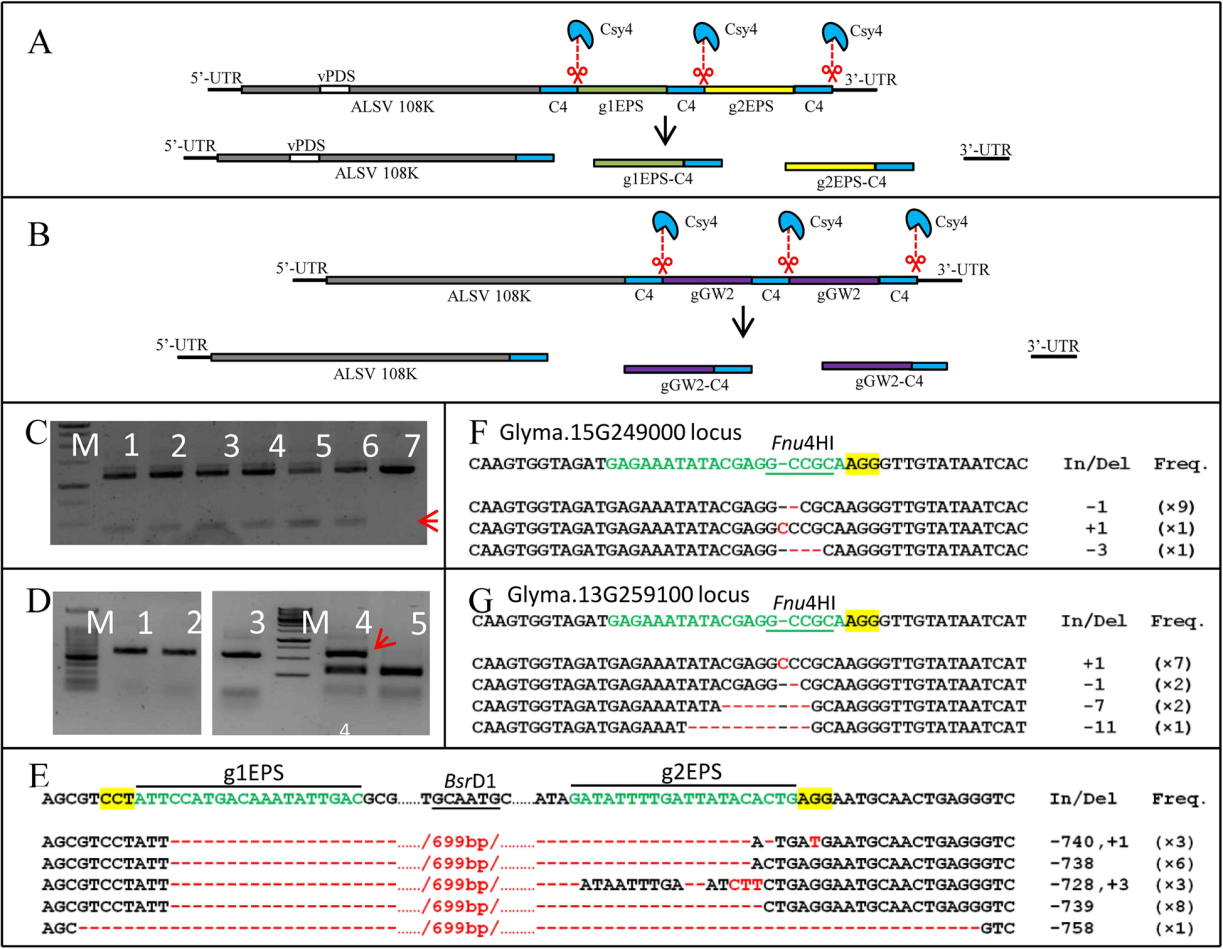
Multiple targeting with CCAC. (**A**) Delivery of multiple gRNAs for multiple targeting with CCAC. g1EPS and were replicated and moved along with ALSV virus RNAs, and released from virus RNAs by Csy4 cleavage. (**B**) Delivery of multiple copies of the same gRNA for single targeting with CCAC. Two copies of were replicated and moved along with ALSV virus RNAs, and released from virus RNAs by Csy4 cleavage. (**C**) DNA gel with PCR amplicons from EPSPS gene. In lane 1 to 6, the PCR templates the genomic DNA extracted from photobleached leaves which inoculated with CCAC carring g1EPS and g2EPS, and infiltrated by Cas9-Cys4 construct, whereas in lane 7 the template was the photobleached leaves without Cas9-Cys4 construct infiltration as control. The red arrow indicated the long fragment deletion of sequence between g1EPS and g2EPS sites. “M” means DNA marker. Uncropped full-length gels of Figure 4C is shown in Supplementary Figure 10. (**D**) Genome editing of GW2 paralogs in soybean hairy root. Lane 1 and lane 3 were the PCR products with hairy root transferred by CCAC carring gGW2s and Cas9-Cys4 construct as template, whereas in lane 2 the template was just transferred by CCAC carring gGW2s as control. Lane 4 and lane 5 were the Fnu4HI digested PCR products from lane 1 and lane 2 respectively. The mutation rate (45.3%) was calculated by ImageJ. The red arrow indicated the Fnu4HI-resistant band. Uncropped full-length gels of Figure 4D is shown in Supplementary Figure 11. (**E**)(**F**)(**G**) Sanger sequencing confirms mutations of EPSPS (E) and GW2 paralogs (F)(G). The red arrow indicated bands in C and D were verified by Sanger sequencing. The results showed indels of EPSPS (**E**) and two GW2 paralogs (**F**)(**G**). The wild type sequence is shown at the top respectively

The ability to edit multiple loci at once using Cas9 can greatly aid in gene function analysis. In order to extend the application of CCAC, the editing potential of multiple targeting with CCAC was tested. We designed two gRNAs (g1EPS and g2EPS; Fig. 4 A) targeting *EPSPS* (*5-enolpyruvylshikimate-3-phosphate synthase*) gene sequence and assembled them in tandem into CCAC. *EPSPS* confers resistance to glyphosate herbicides and has been reported as a gene for Cas9-mediated multiple targeting (Zhang et al. [46]). C4 sites flanked g1EPS and g2EPS in CCAC to release gRNAs from the virus. We agroinfiltrated *N. benthamiana* leaves with gRNAs and a *PDS* fragment (as a visible indicator triggered by VIGS) containing CCAC constructs. Three weeks after infiltration, the upper newly emerging leaves were visibly photobleached by *PDS* VIGS. This indicated the plants were systemically infected by the ALSV carrying the fragments g1EPS and g1EPS. The photobleached leaves were then agroinfiltrated by construct containing *EPSPS* target sequence and Cas9-Csy4 construct. The restriction enzyme site loss method was used to detect Cas9-induced sequence deletion between two gRNA targets (Shan et al. [30]). *Bsr*DI site is present between the two targets in the *EPSPS* sequence (Fig. 4E) and we, therefore, digested the genomic DNA with *Bsr*DI in order to reduce unaltered wild-type DNA and enrich for DNA molecules carrying deletions that removed the *Bsr*DI site. The digested genomic DNA was used to amplify the target fragment, and two PCR bands were detected (Fig. 4 C, Additional file 2 Figure S3). The sequencing results showed the small band was PCR amplicons from the DNA molecules carrying sequence deletion between g1EPS and g2EPS target sites (Fig. 4E). These results showed that CCAC can be used for the multiplex delivery of gRNAs in Cas9-induced genome editing.

### Genome editing with CCAC in soybean

To test the application of CCAC in soybean cells, we used it to carry the gRNA targeting the soybean endogenous *GW2* genes. *GW2* is an important agronomic trait gene that controls seed width and weight, originally cloned from rice (Liang et al. [25]; Song et al. [32]), and was reported as a Cas9 target to improve the crop yield in rice. Based on the sequence analysis, we identified two *GW2* paralogs in soybean: Glyma.15G249000 and Glyma.13G259100. They share 94.2 % identity in amino acid sequence. The gRNA (gGW2) was designed to target these two soybean GW2 paralogs at the same time. To improve the abundance of gRNA, two gGW2s, flanked by the C4 site, were assembled in tandem in CCAC (Fig. 4B), and transferred into soybean hairy root culture with Cas9-Csy4 construct through *Agrobacterium rhizogenes* (*A. rhizogenes*) strain K599. PCR-RE analysis showed that a portion of PCR amplicons (45.3 %) from hairy root genomic DNA with primers amplyfying both GW2 paralogs was resistant to *Fnu*4HI (Fig. 4D, Additional file 2 Figure S4), suggesting that *GW2* genes were edited in the transformed soybean roots. The *Fnu*4HI-resistant band was further Sanger sequenced and the results revealed that nucleotide mutagenesis did occur in both GW2 paralogs (Fig. 4 F and G, Additional file 3), suggesting that the CCAC with Cas9 can be used as a genome editing tool in soybean.

## Discussion

### Virus and host plant species

Employing a virus to carry and deliver gRNA is an effective strategy to facilitate the application of CRISPR-Cas9 in plants especially the ones with low transformation rate by reducing the number of transformations. However, for some plant species, application of this method is hampered by the amount of knowledge of their viral pathogens. Many plants lack the proper virus as their delivery tool vector (Alexander et al. [1]). The virus, which can infect the plant SAM (shoot apical meristem) while maintaining fertility with some virus-free seeds in the infected plants, is ideal for gRNA delivery system development. The gRNA delivery system for a particular crop needs to be studied and developed based on the characters of its specific virus-pathogen interaction.

At present, the purpose of plant virus application in CRISPR-Cas9 can be generally divided into two categories. The first category includes the use of the geminivirus-based replicons to improve the abundance of the components of CRISPR-Cas9 including Cas9, gRNA, and/or DNA repair template in order to enhance the gene targeting frequency. To date, at least two geminiviruses, BeYDV (bean yellow dwarf virus) in tobacco (Baltes et al. [4]) and WDV (wheat dwarf virus) in wheat and rice (Wang et al. [38]), have been reported as DNA replicons for expressing Cas9, gRNA, and DNA repair templates. The second category utilizes the virus movement to carry and deliver gRNA to newly-developing tissues via systemic infection (Ali et al. [2, 3]; Cody et al. [9]; Hu et al. [14]; Jiang et al. [17]; Yin et al. [43]). In our strategy of using CCAC, the gRNA was carried and delivered by viral movement. Athough genetic transformation is still required to express Cas9 in the plant, it can reduce the number of transformations needed for functional study of different genes.

ASLV, a highly infectious soybean RNA virus (Li et al. [24]; Yamagishi and Yoshikawa [40]), was chosen for the establishment of the CCAC system due to several effective properties. Firstly, ALSV can infect plant tissues including the SAM (Nakamura et al. [27]; Yamagishi and Yoshikawa [41]). Genome editing mediated by ALSV in the SAM provides the capability of generating homogenous cells instead of mosaic cells in seeds. Secondly, soybean plants infected by ALSV are fertile and the seeds can be harvested from them (Yamagishi and Yoshikawa [40]). Finally, 80 % of the seeds from ALSV-infected plants are virus-free (Yamagishi and Yoshikawa [41]), which would be useful as breeding materials regarding the virus biosafety reasons.

The integrating position of gRNA in the virus genome is an important factor to consider for an efficient delivery system. ALSV has two genomic RNA molecules: ALSV-RNA1 and ALSV-RNA2 (Igarashi et al. [15]; Li et al. [24]). There are two potential positions in the ALSV-RNA2 sequence for cloning a foreign fragment. The first position is between the two artificial Q/G protease cleavage sites, upstream of the Vp25 protein sequence (Fig. 1B, MCS site). The second position is immediately downstream of the translation stop codon of the Vp24 protein. Cloning of the gRNA into the first position will disrupt the translation of the ALSV-RNA2 polyprotein. The second position was therefore chosen for the insertion of the gRNA.

### gRNA released from virus

Additional bases at the end of the gRNA or other modifications can affect the efficiency of Cas9-mediated mutagenesis (Haurwitz et al. [13]). During the gRNA delivery by the virus, the gRNA is integrated into the virus genome and it replicates and moves along with the virus genomic RNA. To release the gRNA from the virus genome, different strategies have been adopted for different viruses. In the DNA virus vector CaLCuV, the DNA promoter U6 was inserted into the CaLCuV genome to drive the expression of gRNA (Yin et al. [43]). In the RNA virus vector TRV, an RNA virus PEBV (pea early browning virus) promoter was integrated into the TRV genome to control the gRNA expression (Ali et al. [2]). The promoter from BSMV itself was used for transcription of BSMV sgRNA (Hu et al. [14]). Here in the CCAC which is derived from the RNA virus ALSV, the DNA promoter U6 would not function in an RNA virus. We did not adopt the RNA virus PEBV promoter in CCAC as there have not been previous studies that have described infection of soybean cells by PEBV. Furthermore, the size of the PEBV promoter is relatively large (192 bp), particularly for the assembly of multiple gRNAs in tandem by the Golden Gate method. In this study, the Csy4 nuclease was used to release the gRNA from CCAC (Fig. 3 A). Csy4 nuclease is a member of the bacterial CRISPR-Cas system that processes CRISPR RNA into small RNAs and has been engineered to process gRNA for Cas9 nuclease (Haurwitz et al. [13]; Tsai et al. [36]). Here we demonstrate that the Csy4-RNA processing system works effectively in a plant virus. There are several advantages to use Csy4 nuclease to release gRNA from the virus vector. Firstly, the C4 site is very small (20 bp) and this is important for the virus cargo capacity, especially in multiple gRNA assembly. Secondly, the expression of Csy4 nuclease can be driven by a constitutive strong promoter (such as 35 S promoter) regardless of the plant species, while the plant-specific U6 promoter or RNA virus PEBV promoter may not function well in some plants (Mueller et al. [26]; Tang et al. [34]). Thirdly, the Cas9-induced mutations are significantly higher when the gRNAs are processed by Csy4 nuclease compared with gRNAs expressed from individual RNAs (Čermák et al. [7]). Surprisingly, it has been reported that TMV could deliver gRNA in Cas9-mediated DNA cleavage without any additional RNA processing element through an unknown mechanism, in which the gRNA was driven by the subgenomic promoter of TMV (Cody et al. [9]). Additional experiments would need to be performed in order to determine whether this novel mechanism could work in CCAC-gRNA processing, in which the gRNA was integrated downstream of a long single open reading frame of ALSV.

### Cas9-induced genome editing with CCAC

In the CCAC system, we inserted gRNA(s) immediately downstream of the ALSV-RNA2 108 K polyprotein stop codon, to not affect the translation of virus proteins (Fig. 1B). It has been previously reported that the modification after the stop codon may reduce the accumulation of ALSV during the initial infection stage leading to low infection efficiency (Kon and Yoshikawa [22]). Therefore, a viral RNA silencing suppressor P19 (Kon and Yoshikawa [22]; Zhang et al. [44]) was co-agroinfitrated to improve the initial CCAC infection. Furthermore, we cloned a 102 bp *PDS* fragment into the MCS site of CCAC for inducing VIGS (Fig. 2 A). The photobleaching phenotype of PDS VIGS (Velásquez et al. [37]) was used as a visible indicator of virus infection (Fig. 2B). We could therefore easily distinguish and select the infected plants for further experiments.

In this study, we targeted an endogenous locus PDS (Fig. 3) and an exogenous EPSPS DNA fragment in *N. benthamiana*, as well as two endogenous GW2 paralogs in soybean (Fig. 4). All of the loci tested were edited effectively with the CCAC system. The Cas9 and Csy4 nucleases were transiently expressed for these targets. We hypothesize that the stable expression of the nucleases would improve the editing efficiency (Ali et al. [2]; Yin et al. [43]), so further experiments developing a stable transformation system in soybean to optimize and extend the CCAC application are underway. We also tested the off-target activity by a previously described method (Nekrasov et al. [28]). The results showed there was no off-target activity of gPDS from CCAC (Additional file 1 Figure S1). Although more analyses are required to evaluate comprehensive off-target cleavage, we reasoned that the off-target activity directed by gRNA from CCAC and traditional U6-driven gRNA may be the same, as the gRNA sequences released from CCAC and expressed by U6 are the same. Small indels (Figs. 3D and 4 F and G) and larger deletion (Fig. 4E) were generated with CCAC. We did not test HDR-mediated nucleotide substitution, in which a DNA repair template is typically required. Although the RNA virus-based vector including CCAC cannot be used to carry the DNA sequence, several reports indicate that RNA also can act as a template for DNA double-stranded break repair (Keskin et al. [20]; Shen et al. [31]; Yang and Qi [42]). Therefore, the CCAC and other plant RNA viruses could be used to carry RNA repair template for Cas9-induced nucleotide substitution in plants. This is a very important topic for future work.

The CCAC can facilitate the application of CRISPR-Cas9 in soybean gene function studies with the following strategy. Firstly, the Cas9-Csy4 construct is delivered into soybean by regular *Agrobacterium*-mediated transformation to obtain a stable transgenic line. Then the target-specific gRNA is cloned into CCAC and delivered into the transgenic line by virus infection for genome editing. In this way, a stable transformation is only performed once to transform Cas9 and Csy4. Once the Cas9 and Csy4 expressing stable transgenic line is prepared, the target-specific gRNA can be delivered into soybean on a large-scale and with high-efficiency by a viral infection. This avoids having to repeat the time-consuming and low-efficiency stable transformation due to the change in target.

## Conclusions

Taken together, the CCAC can not only be used in single targeting for short indels mutation but also in multiple targeting for large fragment deletion. It provides a fast and efficient method to deliver gRNA(s) for Cas9-induced mutagenesis, which is very practical in soybean gene analysis and trait improvement via CRISPR-Cas9 genome editing.

## Methods

### Plasmid construction

The primers used are listed in Additional file 1 Figure S3. To construct an ALSV-RNA1-based vector, an ALSV-RNA1 expression cassette containing 35 S promoter, ALSV-RNA1 sequence, and nos terminator was amplified from pEALSR1 (Igarashi et al. [15]) using primers M13-attB1-F0 and M13-attB2-R0 (Additional file 1 Figure S2A). The PCR products were inserted into pDONR207 by BP reaction to produce p207ALR1 and then transferred to the binary vector pEarleyGate301 to generate the binary plasmid p301ALR1.

In order to construct the ALSV-RNA2-based vector, an overlapping PCR technique was applied to introduce two *Bsa*I sites and one C4 site (Additional file 1 Figure S2B). In the first round of PCR, fragment 1 was amplified using the primers ALR2-*Xho*I-F and ALR2-*Bsa*I-R with pEALSR2L5R5 as template; fragment 2 was amplified using the primers ALR2-*Bsa*20-F and ALR2-*Spe*I-R2 with pEALSR2L5R5 as a template. In the second round of PCR, the overlapping PCR was performed using primers ALR2-*Xho*I-F and ALR2-*Spe*I-R2 with gel-purified fragment 1 and fragment 2 as templates. The PCR products were digested by *Xho*I and *Spe*I and then inserted into the same digested pEALSR2L5R5 (Igarashi et al. [15]), producing pEALSR2-*Bsa*I-C4. One of the *Bsa*I sites in pDONR207 was eliminated by site-directed mutagenesis with primers 207-*Bsa*Imut-F and 207-*Bsa*Imut-R, producing p207m. The ALSV-RNA2 expression cassette including 35 S promoter, ALSV-RNA2, and nos terminator was amplified from pEALSR2-*Bsa*I-C4 using primers M13-attB1-F0 and M13-attB2-R0. The generated PCR product was inserted into p207m by BP reaction to produce p207ALR2 and then transferred to pEarleyGate301 to generate the binary plasmid p301ALR2 by LR reaction.

The constructs of ALSV-RNA2-based derivatives, including p301ALR2-vPDS (for PDS VIGS), p301ALR2-vPDS-gPDS (for genome editing of PDS), p301ALR2-vPDS-gEPS1-gEPS2 (for genome editing of EPSPS), and p301ALR2-2gGW2 (for genome editing of GW2) are described in Additional file 1 Figure S2 with the sequences presented in Additional file 1 Figure S4. The plasmid pEG104-Cas9-P2A-Csy4 (Cas9-Csy4 construct) was constructed by Golden Gate assembly with six module plasmids (Additional file 1 Figure S5). The details of constructs and sequences are described in Additional file 1 Figure S6 and S7. To construct the targeting plasmid containing the EPSPS sequence, EPSPS fragment was amplified from common bean (*Phaseolus vulgaris*) cDNA by primer EPSPS-F and EPSPS-R. The PCR product was inserted into pDONR207 by BP reaction and subsequently transferred to pEarleyGate301 by LR reaction to generate p301-EPSPS (Additional file 1 Figure S2C). To express the RNA silencing suppressor, P19 was amplified by primers P19-F and P19-R with pBPMV-P19 (Zhang et al. [44]) astemplate. The PCR products were inserted into pDONR207 by BP reaction and transferred to pEarleyGate201 by LR reaction to generate p201-P19. The ALSV isolates were obtained by a Materials Transfer Agreement between Iwate University and Agriculture and Agri-Food Canada, and an import permit granted by the Canadian Food Inspection Agency (P-2013-02404). All research material was managed according to the requirements of Plant pest containment level 1 as described in the Containment Standards for Facilities Handling Plant Pests (plant compliance number: PC-2013-032.

### Golden Gate assembly and Gateway transfer

For Golden Gate assembly, the PCR products of gRNA fragment(s) were inserted into the p207ALR2-based vector for the construction of gRNA carrying ALSV plasmids, whereas the six-module plasmids were inserted into the recipient vector pGGZ001 for construction of pEG104-Cas9-P2A-Csy4. The Golden Gate cloning reaction was performed as previously described (Lampropoulos et al. [23]) with some modification. Briefly, 0.5 µl of gel-purified PCR product(s) or 0.5 µl each of the modules (150 ng/µl each) were mixed with 0.5 µl vector (150 ng/µl), 0.5 µl T4 ligase buffer, 0.3 µl *Bsa*I-HF (NEB, R3535L), 0.3 µl T4 ligase (NEB, M0202M), and 0.5 µl T4 ligase buffer to a total volume of 5 µl. The reaction was performed for 50 cycles of 37 °C for 5 min and 16 °C for 5 min each, followed by 50 °C for 5 min and 80 °C for 10 min. For gRNA carrying ALSV plasmids, 0.5 µl *Bsa*I-HF was added and the reaction incubated at 37 °C for 1 h to cut the unassembled p207ALR2-based vector. The reaction was then transferred to DH5α competent cells for cloning and screening. The DNA fragments assembled into p207ALR2-based or pGGZ001 were transferred to binary vector by LR recombination reaction. The LR reaction followed the manufacturer’s instruction of LR Clonase II Enzyme Mix (Invitrogen, 11791-020).

### Sequence analysis

For GW2 homology analysis, the amino acid sequence of rice GW2 (GenBank: EF447275) was used to Blast the soybean database *Glycine max* Wm82.a2.v1 at Phytozome v12.1. The identity analysis of the GW2 paralogs was based on the “protein homologs” link at the Phytozome v12.1 (https://phytozome.jgi.doe.gov/pz/portal.html).

### Virus infitration and gene expression infiltration of ***N. benthamiana***

The binary plasmids were introduced into *A. tumefaciens* GV3101 by electroporation for expression in *N. benthamiana*. Agroinfitration was carried out as previously described (Kon and Yoshikawa [22]) with some modifications. Briefly, *A. tumefaciens* cultures were grown in LB liquid medium at 30 °C. Cells were pelleted and washed twice with infiltration buffer (10 mM MgCl_2_, 100 µM acetosyringone), then resuspended in infiltration buffer to a final OD_600_ = 0.5. The viral RNA silencing suppressor P19 was always co-agroinfitrated with virus constructs. *A. tumefaciens* carrying p201-P19, p301ALR1, and p301ALR2 or their derivatives were mixed at a 1:1 ratio, incubated for 2 h, and then infiltrated onto the abaxial leaf surface using a 1-ml syringe. For virus rub-inoculation, *N. benthamiana* leaves with symptoms were homogenized with inoculation buffer (0.1 M Tris-HCl pH 7.5, 0.1 M NaCl, 50 mM MgCl_2_) in a mortar and pestle, then mechanically inoculated onto leaves of *N. benthamiana* as previously described (Igarashi et al. [15]). For the Cas9-Csy4 construct, *A. tumefaciens* containing pEG104-Cas9-P2A-Csy4 were infiltrated into plant leaf surface of which one-third of the area had been photobleached by VIGS-mediated PDS silencing. The fluorescent signal were observed using Leica confocal microscope TCS SP2 imaging at 2 dpi.

### Soybean hairy root transformation

The plasmids were introduced into *A. rhizogenes* K599 via electroporation for soybean hairy root transformation. The hairy root transformation was performed as previously described (Jian et al. [16]; Kereszt et al. [19]) with some modifications. Briefly, a single *A. rhizogenes* colony was suspended in 5 ml liquid LB medium for growing overnight at 30 °C and 20 µl of culture was sub-cultured in 200 ml liquid LB medium for growing 16 h at 30 °C. Cells were pelleted and resuspended with liquid LB to OD_600_ = 0.5. Williams 82 soybean (*Glycine max*) cultivar was used for *A. rhizogenes*-mediated transformation. A total of 45 seeds were tested per construct in three separate experiments. The seeds were sterilized according to (Khandual and Reddy [21]) and plated on Gamborg B5 Basal Medium (PhytoTechnology Laboratories, G398) for 10 days in the dark or until germination or the emergence of the radicle. The emerging root was removed using a sterile scalpel blade and the remaining intact seed was used as explant for *A. rhizogenes*-mediated transformation. The explants were submerged in prepared *A. rhizogenes* culture (OD_600_ = 0.5) for 30 min at room temperature and then dried on sterile filter paper for about 30 min. Dried explants were then transferred to ½ MS (Murashige & Skoog Modified Basal Medium with Gamborg Vitamins [PhytoTechnology Laboratories, M404]) media, containing 2 % sucrose and 0.8 % agar (pH 5.4). Petri plates were covered with filter paper for co-cultivation in the dark. After 3 days of co-cultivation, the explants were washed three times with sterile water containing 300 µg/L timentin and transferred to a solid ½ MS medium containing 2 % sucrose, 0.8 % agar and 250 mg/L cephalexin (pH 5.7) for hairy root induction. Prior to transfer to new ½ MS plates, the seed coat was removed using forceps. Hairy roots were allowed to develop under light conditions at room temperature in sealed Petri plates. The hairy roots were collected after 25 days either for immediate DNA extraction or frozen in liquid nitrogen and stored at -80 °C for future use.

### RNA and DNA analysis

Total RNA was extracted from plants using Plant/Fungi Total RNA Purification Kit (Norgen, 31,350). cDNA was synthesized using 5X iScript cDNA Synthesis Kit (Bio-Rad, 1,708,890). DNA was extracted using the DNeasy Plant Mini Kit (Qiagen, 69,106). Standard PCR was performed with 2X PCR Taq MasterMix with dye (abm, G013-dye) while high-fidelity PCR was performed using Phusion High-Fidelity DNA Polymerase (NEB, M0530S).

### Detection of mutations in genomic DNA

Cas9-induced DNA mutagenesis was detected by PCR-RE as previously described (Nekrasov et al. [28]) with some modifications. In brief, high-fidelity PCR was performed using genomic DNA with or without restriction enzyme digestion as a template. PCR primers flanking the targeting site(s) are shown in Additional file 1 Figure S3. The PCR amplicons were digested with the corresponding restriction enzyme and analysed by agarose gel electrophoresis. The expected bands were gel-purified and inserted into pGGZ001 by Golden Gate assembly. Primers Z001-F and Z001-R were used for Sanger sequencing to detect the potential mutations of the inserted DNA fragments. The mutation rate was measured by ImageJ and calculated by dividing the intensity of the uncut band by the intensity of all bands in the lane. All the genomic editing assays were repeated independently at least three times using different plants. The off-target assay was performed as previously described (Nekrasov et al. [28]). Nine potential off-targets (Additional file 1 Figure S1), which amplified a PCR product using the *Mly*I-digested genomic DNA, were tested in this project.

## Supplementary Information

**Additional File 1: Figure S1.** Assays of potential off-targets. **Figure S2.** Diagram of ALSV-based vectors construction. **Figure S3.** Primers used in this project. **Figure S4.** BsaI cloning site sequence in p207ALR2 derivatives. **Figure S5.** Six module plasmids for pGGZ001-Cas9-P2A-Csy4 construction. **Figure S6.** Diagram and expression of Cas9-Csy4 construct. **Figure S7.** BsaI cloning site sequence in plasmid pGGZ001-Cas9-P2A-Csy4.

**Additional File 2:** Original uncropped gel pictures **Figure S1.** Uncropped full-length gels of Figure 2C. **Figure S2.** Uncropped full-length gels of Figure 3C. **Figure S3.** Uncropped full-length gels of Figure 4C. **Figure S4.** Uncropped full-length gels of Figure 4D.

**Additional File 3.** Sanger sequencing raw data The Sanger sequencing raw data of genomic editing, including PDS gene, EPSPS gene and GW2 gene.

## Data Availability

All data generated or analysed during this study are included in this published article and its supplementary information files. The datasets used and/or analysed during the current study are available from the corresponding author on reasonable request.

## Abbreviations

CRISPR/Cas9: Clustered Regularly Interspaced Short Palindromic Repeats/ CRISPR-associated 9.
gRNAs: guide RNAs.
ALSV: Apple latent spherical virus.
PDS: *phytoene desaturase*.
VIGS: virus-induced gene silencing.
CCAC: Cas9 based Csy4-processed ALSV Carry System.
