# Supplementary material for: Development of a Csy4-processed guide RNA delivery system with soybean-infecting virus ALSV for genome editing
Source: BMC Plant Biol. 2021 Sep 13;21:419. doi: 10.1186/s12870-021-03138-8 (PMC8436479; doi:10.1186/s12870-021-03138-8)
Supplement: Supplementary file 1 — Additional File 1: Figure S1. Assays of potential off-targets. Figure S2. Diagram of ALSV-based vectors construction. Figure S3. Primers used in this project. Figure S4. BsaI cloning site sequence in p207ALR2 derivatives. Figure S5. Six module plasmids for pGGZ001-Cas9-P2A-Csy4 construction. Figure S6. Diagram and expression of Cas9-Csy4 construct. Figure S7. BsaI cloning site sequence in plasmid pGGZ001-Cas9-P2A-Csy4. [file 12870_2021_3138_MOESM1_ESM.docx]

**Additional file 1**


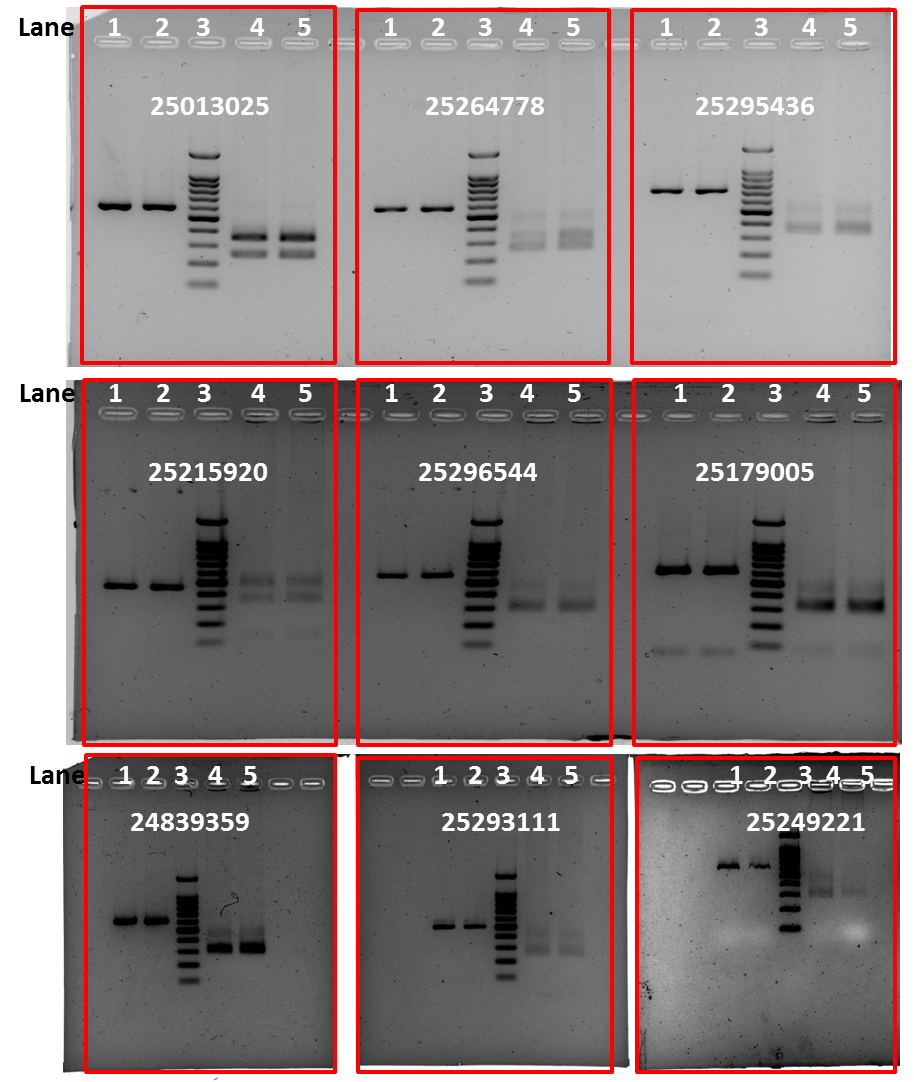


**Figure S1. Assays of potential off-targets.**

Twelve off-target loci were choice to be amplified with the indicated primer pairs. The *Mly*I digested genomic DNA extracted from photobleached *N. Benthamiana* leaves which infected by p301ALR1 together with p301ALR2-vPDS-gPDS, and infiltrated (as “Cas9”) or not infiltrated (as “control”) with pEG104-Cas9-P2A-Csy4 were used as PCR template. Out of the 12 locus, 9 gave a PCR band of expected size. The locus 25287026, 25269131 and 25159671 did not get band. These 9 PCR bands were further digested by *Mly*I and run agarose gel. The locus number was shown in the top of each gel panel. In each gel panel, lane 1 and lane 2 are PCR amplicons from *Mly*I digested genomic DNA of “Cas9” and “control” respectively, lane 3 are DNA Marker, lane 4 and lane 5 are *Mly*I digested PCR products from lane 1 and lane 2.


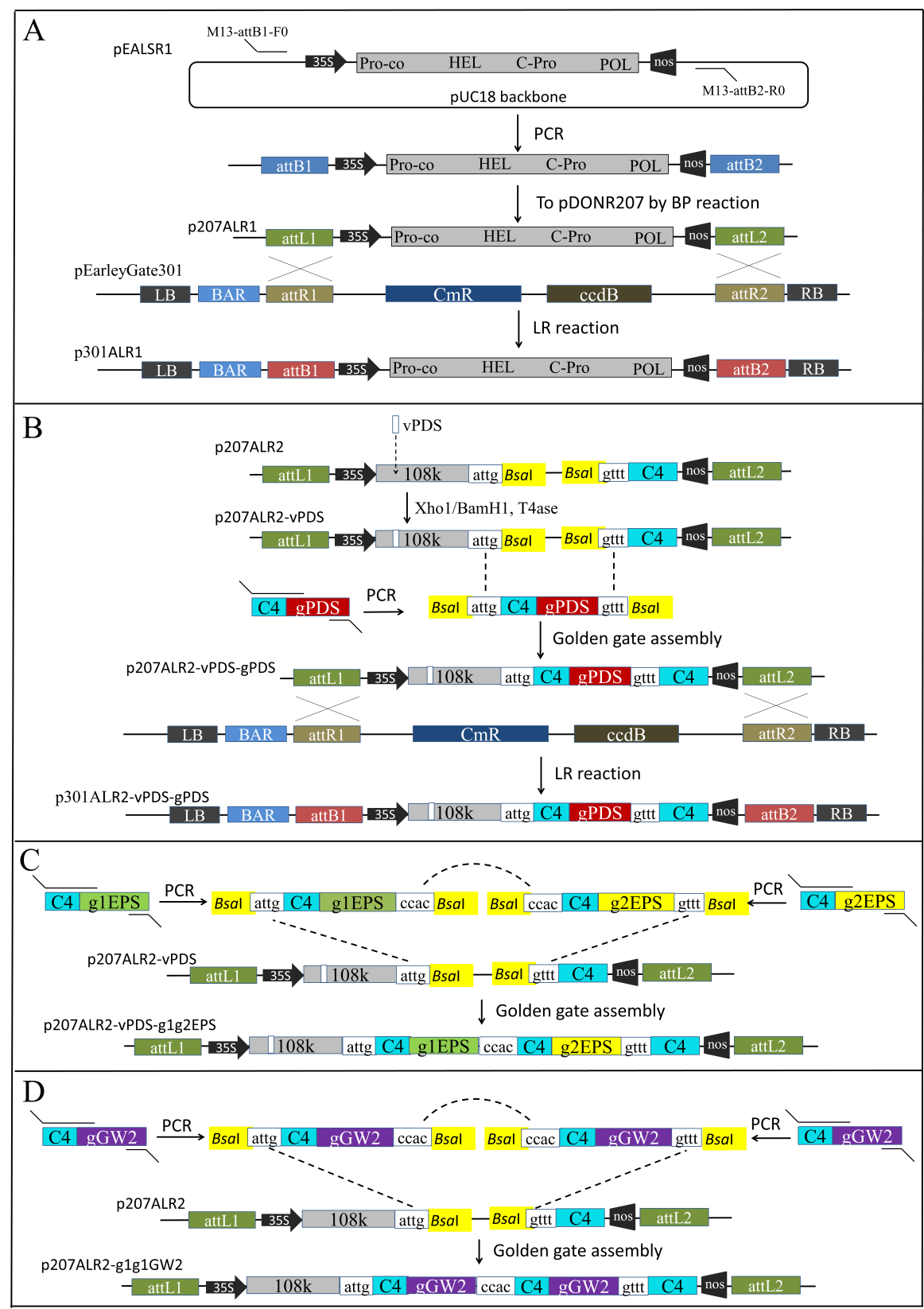


**Figure S2. Diagram of ALSV-based vectors construction.**

(A) ALSV-RNA1 binary vector p301ALR1 construction. ALSVRNA1 expression cassette was cloned from pUC18-based plasmid into the binary vector pEarleyGate301 to generate p301ALR1,

(B) Construction of p301ALR2-vPDS-gPDS. PDS fragments were amplified using primers NbPDSVG2- *Xho*I-F0 and NbPDSVG2-*Bam*HI-R0 with *N. benthamiana* cDNA as template. PCR products were digested by *Xho*I and *Spe*I and were inserted into the same digested p207ALR2, to generate p207ALR2-vPDS. gPDS fragments were amplified using primers gPDS-20(MF)-F0 and gRNA-(MR)-R with plasmid pHSN6A01 as template. The PCR fragments were then inserted into p207ALR2-vPDS by Golden gate method to produce p207ALR2-vPDS-gPDS. The LR reaction was performed using p207ALR2-vPDS-gPDS and pEarleyGate301 to create binary plasmid p301ALR2-vPDS-gPDS.

(C) Construction of gRNA plasmid with multiple gRNAs for multiple targeting. g1EPS and g2EPS fragments, which have different 20bp gRNA spacer sequence, were amplified using primers g1EPS-20(MF)-F0 and gRNA-(c1)-R, g2EPS-20(c1)-F0 and gRNA-(MR)-R respectively. Then they were assembled into p207ALR2-vPDS by Golden gate method to produce p207ALR2-vPDS-gEPS1-gEPS2.

(D) Construction of gRNA plasmid with multiple copies of the same gRNA for single targeting. Two gGW2 gRNA fragments with the same 20bp gRNA spacer sequence and different *Bsa*I overhangs were amplified with primers gGW2-(MF)-F0 and gRNA-(c1)-R, gGW2-(c1)-F0 and gRNA-(MR)-R respectively. Then they were assembled into p207ALR2 by Golden gate method to produce p207ALR2-2gGW2.

The 4bp nuclear acids written in box are *Bsa*I restriction overhangs created during Golden gate assembly. The ALSVRNA2 expression cassette of all p207ALR2-based constructs can be transferred into pEarleyGate301 to create p301ALR2 based binary constructs.

| Primer name | Sequence (5’-3') | Note |
| --- | --- | --- |
| M13-attB1-F0 | GGGGACAAGTTTGTACAAAAAAGCAGGCTTCCCCAATACTTGTcTGGcACCTGTTTTCCCAGTCACGAC | underline stands attB1 sequence, wavy line stands M13F sequence |
| M13-attB2-R0 | GGGGACCACTTTGTACAAGAAAGCTGGGTCCCCAATACTTGTATGGcATACCAGGAAACAGCTATGAC | underline stands attB2 sequence, wavy line stands M13R sequence |
| ALR2-*Xho*I-F | ccgCTCGAGCCCGGGGGATCC | underline stands *Xho*I site |
| ALR2-*Bsa*I-R | agtgaacAAACtGAGACCtgttGGTCTCaCAATctaggtgtaaccagct | two underlines stand two *Bsa*I recognition sites, two boxes stand two overhangs created by *Bsa*I |
| ALR2-*Bsa*20-F | caGGTCTCaGTTTgttcactgccgtataggcagtttggtctttctgcta | underline stands *Bsa*I recognition sites, box stands overhang created by *Bsa*I, wavy line stands C4 site |
| ALR2-*Spe*I-R2 | Gactagttcaaaagaaagagaaaaagaaaaggactcaaagatagcagaaagaccaaactgcctatac | underline stands *Spe*I site |
| 207-*Bsa*Imut-F | CGCAAGGTTTCGCTCTCCACGCATC | underline stands mutated *Bsa*I site |
| 207-*Bsa*Imut-R | GATGCGTGGAGAGCGAAACCTTGCG |  |
| NbPDSVG2- *Xho*I-F0 | CCGctcgagAACCCTCCTGAGAGACTTTG | underline stands *Xho*I site |
| NbPDSVG2-*Bam*HI-R0 | CGggatccCTCATTCAGCTCGATC | underline stands *Bam*HI site |
| gPDS-20(MF)-F0 | aacaGGTCTCaATTGgttcactgccgtataggcagGCCGTTAATTTGAGAGTCCAGTTTTAGAGCTAGAAATA | underline stands *Bsa*I recognition sites, box stands overhang created by *Bsa*I, wavy line stands C4 site, double line stands 20bp gRNA spacer sequence, dashed line stands gRNA scaffold special sequence |
| gRNA-(MR)-R | aacaGGTCTCaAAACGCACCGACTCGGTGCCA | underline stands *Bsa*I recognition sites, box stands overhang created by *Bsa*I, dashed line stands gRNA scaffold special sequence |
| g1EPS-20(MF)-F0 | aacaGGTCTCaATTGgttcactgccgtataggcagGTCAATATTTGTCATGGAATGTTTTAGAGCTAGAAATA | underline stands *Bsa*I recognition sites, box stands overhang created by *Bsa*I, wavy line stands C4 site, double line stands 20bp gRNA spacer sequence, dashed line stands gRNA scaffold special sequence |
| g2EPS-20(c1)-F0 | aacaGGTCTCaccacgttcactgccgtataggcagGATATTTTGATTATACACTGGTTTTAGAGCTAGAAATA | underline stands *Bsa*I recognition sites, box stands overhang created by *Bsa*I, wavy line stands C4 site, double line stands 20bp gRNA spacer sequence, dashed line stands gRNA scaffold special sequence |
| gRNA-(c1)-R | aacaGGTCTCagtggGCACCGACTCGGTGCCA | underline stands *Bsa*I recognition sites, box stands overhang created by *Bsa*I, dashed line stands gRNA scaffold special sequence |
| gGW2-(MF)-F0 | aacaGGTCTCaATTGgttcactgccgtataggcagGAGAAATATACGAGGCCGCAGTTTTAGAGCTAGAAATA | underline stands *Bsa*I recognition sites, box stands overhang created by *Bsa*I, wavy line stands C4 site, double line stands 20bp gRNA spacer sequence, dashed line stands gRNA scaffold special sequence |
| gGW2-(c1)-F0 | aacaGGTCTCaccacgttcactgccgtataggcagGAGAAATATACGAGGCCGCAGTTTTAGAGCTAGAAATA | underline stands *Bsa*I recognition sites, box stands overhang created by *Bsa*I, wavy line stands C4 site, double line stands 20bp gRNA spacer sequence, dashed line stands gRNA scaffold special sequence |
| A10D-F | tactcgatcggcctcgAcattgggactaactctg | Cas9 site-directed mutagenesis primer |
| A10D-R | cagagttagtcccaatgTcgaggccgatcgagta | Cas9 site-directed mutagenesis primer |
| A840H-F | gattacgacgtcgatCAcatcgttccacagtcattc | Cas9 site-directed mutagenesis primer |
| A840H-R | gaatgactgtggaacgatgTGatcgacgtcgtaatc | Cas9 site-directed mutagenesis primer |
| EF1a-F | AAGGTCCAGTATGCCTGGGTGCTTGAC | RT-PCR primer of control gene EF1a |
| EF1a-R | AAGAATTCACAGGGACAGTTCCAATACCA | RT-PCR primer of control gene EF1a |
| ALSV-RNA1-F | cctttaacgttgaacatg | RT-PCR primer to detect ALSV-RNA1 |
| ALSV-RNA1-R | tctcgcaatgcttgatcc | RT-PCR primer to detect ALSV-RNA1 |
| F1 | GCGAGGCACTCCTTA | RT-PCR primer to detect ALSV-RNA2 |
| R1 | CTCATTCAGCTCGATC | RT-PCR primer to detect “vPDS” fragment in ALSV-RNA2 construct |
| R2 | gcaaggtggtcgtga | RT-PCR primer to detect ALSV-RNA2 |
| F3 | gtgggaagtttgattacc | RT-PCR primer to detect ALSV-RNA2 |
| R3 | GCACCGACTCGGTGCCA | RT-PCR primer to detect gRNA sequence in ALSV-RNA2 construct |
| F5 | ATGGtgagcaagggcgaggag | RT-PCR primer to detect YFP-Cas9 expression |
| R5 | tcgtagggtacttctcgtg | RT-PCR primer to detect YFP-Cas9 expression |
| F6 | ttccacaggctggaggag | RT-PCR primer to detect Cas9 expression |
| R6 | tccgacagattcttggcag | RT-PCR primer to detect Cas9 expression |
| F7 | aagtatgtgaacttcctc | RT-PCR primer to detect Cas9-Csy4 expression |
| R7 | tcagaaccagggcacga | RT-PCR primer to detect Cas9-Csy4 expression |
| 25215920F | TTGGTCCTAAGTACAATAGCTGGT | off-target primer to detectNiben.v0.3.Scf25215920 locus |
| 25215920R1 | TCGCTCCTTGTGTCAACTTCA | off-target primer to detectNiben.v0.3.Scf25215920 locus |
| 25013025F | GGGTTGGCAGTTACAGTTGAA | off-target primer to detect Niben.v0.3.Scf25013025 locus |
| 25013025R | GTGTTGAAGGCGTTGGAGAT | off-target primer to detect Niben.v0.3.Scf25013025 locus |
| 25296544F | AAGAGAGGTCCCCTCCATTT | off-target primer to detect Niben.v0.3.Scf25296544 locus |
| 25296544R | CAAAGGCGGAGCTACAAAAA | off-target primer to detect Niben.v0.3.Scf25296544 locus |
| 25295436F | TCAATGTTCTTGGGGGAAAG | off-target primer to detect Niben.v0.3.Scf25295436 locus |
| 25295436R | CCAGCCTTGGTCTGTCCATA | off-target primer to detect Niben.v0.3.Scf25295436 locus |
| 25293111F | GAGATCTTTATGTTTGGACATGTGA | off-target primer to detect Niben.v0.3.Scf25293111 locus |
| 25293111R1 | TCATTATATCAGAGCTGGTATCT | off-target primer to detect Niben.v0.3.Scf25293111 locus |
| 25287026F | TGTGAAAAGAAACTAGCTAACTGGAA | off-target primer to detect Niben.v0.3.Scf25287026 locus |
| 25287026R1 | CTCCATATGGCCCTCTTACAG | off-target primer to detect Niben.v0.3.Scf25287026 locus |
| 25269131F | CCATAGTGTTGAGCCCATGA | off-target primer to detect Niben.v0.3.Scf25269131 locus |
| 25269131R1 | TTATGGTTTTCACTAATATTATGCA | off-target primer to detect Niben.v0.3.Scf25269131 locus |
| 25264778_2F | CTTCCGGGATCGAATCAATA | off-target primer to detect Niben.v0.3.Scf25264778 locus |
| 25264778_2R | AATTCAAATCAAAGGTGGGAAT | off-target primer to detect Niben.v0.3.Scf25264778 locus |
| 25249221F | CTGTATGGCGCCAGCTTT | off-target primer to detect Niben.v0.3.Scf25249221 locus |
| 25249221R | TGTTGAGCTTAGCTTCCATTG | off-target primer to detect Niben.v0.3.Scf25249221 locus |
| 25179005F | CGCGACTTTCTGGATGATAA | off-target primer to detect Niben.v0.3.Scf25179005 locus |
| 25179005R | GCTTCGTCGTGTGGTAAGTTC | off-target primer to detect Niben.v0.3.Scf25179005 locus |
| 25159671F | CTTCCGGGATCGAATCAATA | off-target primer to detect Niben.v0.3.Scf25159671 locus |
| 25159671R | TTCAAATCATCGGTGGGAGT | off-target primer to detect Niben.v0.3.Scf25159671 locus |
| 24839359F | ATCAGACCTTGGGGGTACAA | off-target primer to detect Niben.v0.3.Scf24839359 locus |
| 24839359R | GGTTAGTTCACTGATCGTCTAAGG | off-target primer to detect Niben.v0.3.Scf24839359 locus |
| PDS-(A)-F | aacaGGTCTCaACCTGCTTTGCTTGAGAAAAGCTCTC | forward primer flanking the gPDS site. underline stands *Bsa*I recognition sites, box stands overhang created by *Bsa*I |
| PDS-(G)-R | aacaGGTCTCaATACACATAACAAATTCCTTTGCAAGC | reverse primer flanking the gPDS site. underline stands *Bsa*I recognition sites, box stands overhang created by *Bsa*I |
| EPS-(A)-F | aacaGGTCTCaACCTTGCCAGGGTCTAAGTCTC | forward primer flanking the g1EPS and g2EPS sites. underline stands *Bsa*I recognition sites, box stands overhang created by *Bsa*I |
| EPS-(G)-R | aacaGGTCTCaATACCACAATCAACATCTGCAC | reverse primer flanking the g1EPS and g2EPS sites. underline stands *Bsa*I recognition sites, box stands overhang created by *Bsa*I |
| GW2-(A)-F | aacaGGTCTCaACCTTGCAGGGATTTCGGGATG | forward primer flanking the gGW2 site. it can anneal to two GW2 paralogs . underline stands *Bsa*I recognition sites, box stands overhang created by *Bsa*I |
| GW2-(G)-R | aacaGGTCTCaATACCAAATTTGCAGTTTGAAA | reverse primer flanking the gGW2 site. it can anneal to two GW2 paralogs . underline stands *Bsa*I recognition sites, box stands overhang created by *Bsa*I |
| Z001-F | GCGATCGCaccaGGTACC |  |
| Z001-R | gtGCATGCttggTCTAGA |  |
| P19-F | GGGGACAAGTTTGTACAAAAAAGCAGGCTTCATGGAACGAGCTATACAAG |  |
| P19-R | GGGGACCACTTTGTACAAGAAAGCTGGGTCCTCGCTTTCTTTTTCGAAG |  |
| EPSPS-F | GGGGACAAGTTTGTACAAAAAAGCAGGCTTCTGCCAGGGTCTAAGTCTC |  |
| EPSPS-R | GGGGACCACTTTGTACAAGAAAGCTGGGTCCACAATCAACATCTGCAC |  |

**Figure S3. Primers used in this project.**


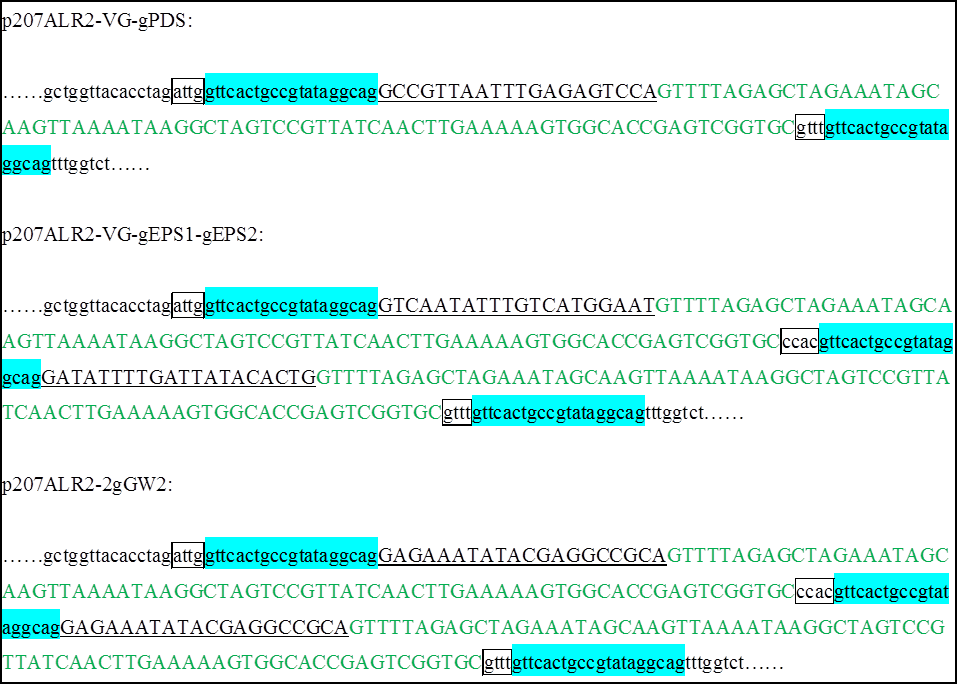


**Figure S4. *Bsa*I cloning site sequence in p207ALR2 derivatives.**

C4 site highlighted in blue, gRNA backbone sequence colored in green. 20bp target sequence underlined. The sequences in small font with ellipsis (……) at the 5’ and 3’ ends are partial of ALSV-R2 108K protein coding sequence and 3’-UTR sequence respectively. *Bsa*I restriction overhangs created during Golden gate assembly written in box.

**
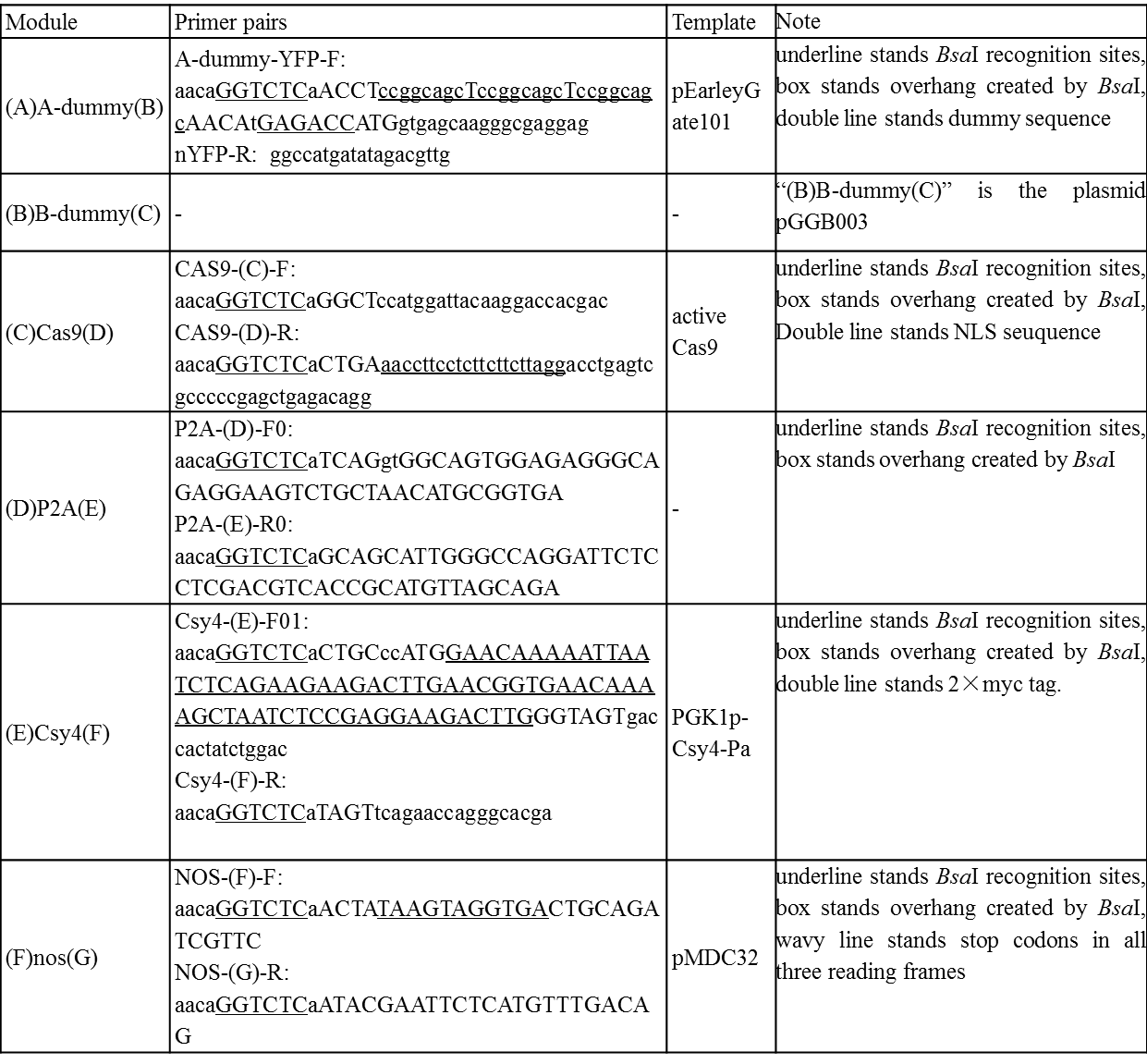
**

**Figure S5. Six module plasmids for pGGZ001-Cas9-P2A-Csy4 construction.**

The PCR was performed using indicated template and primers. Then the PCR products were introduced into pGEM-T easy vector by TA cloning, to produce the corresponding module plasmid. To create “(C)Cas9(D)”, the inactive Cas9 coding sequence at plasmid pHSN6A01 was mutant to “active Cas9” (A10D and A840H) through site-directed mutagenesis with primers A10D-F and A10D-R, A840H-F and A840H-R. To create “(D)P2A(E)”, PCR was performed using primers P2A-(D)-F0 and P2A-(E)-R0, no template required since the two primers were overlapped by themselves.


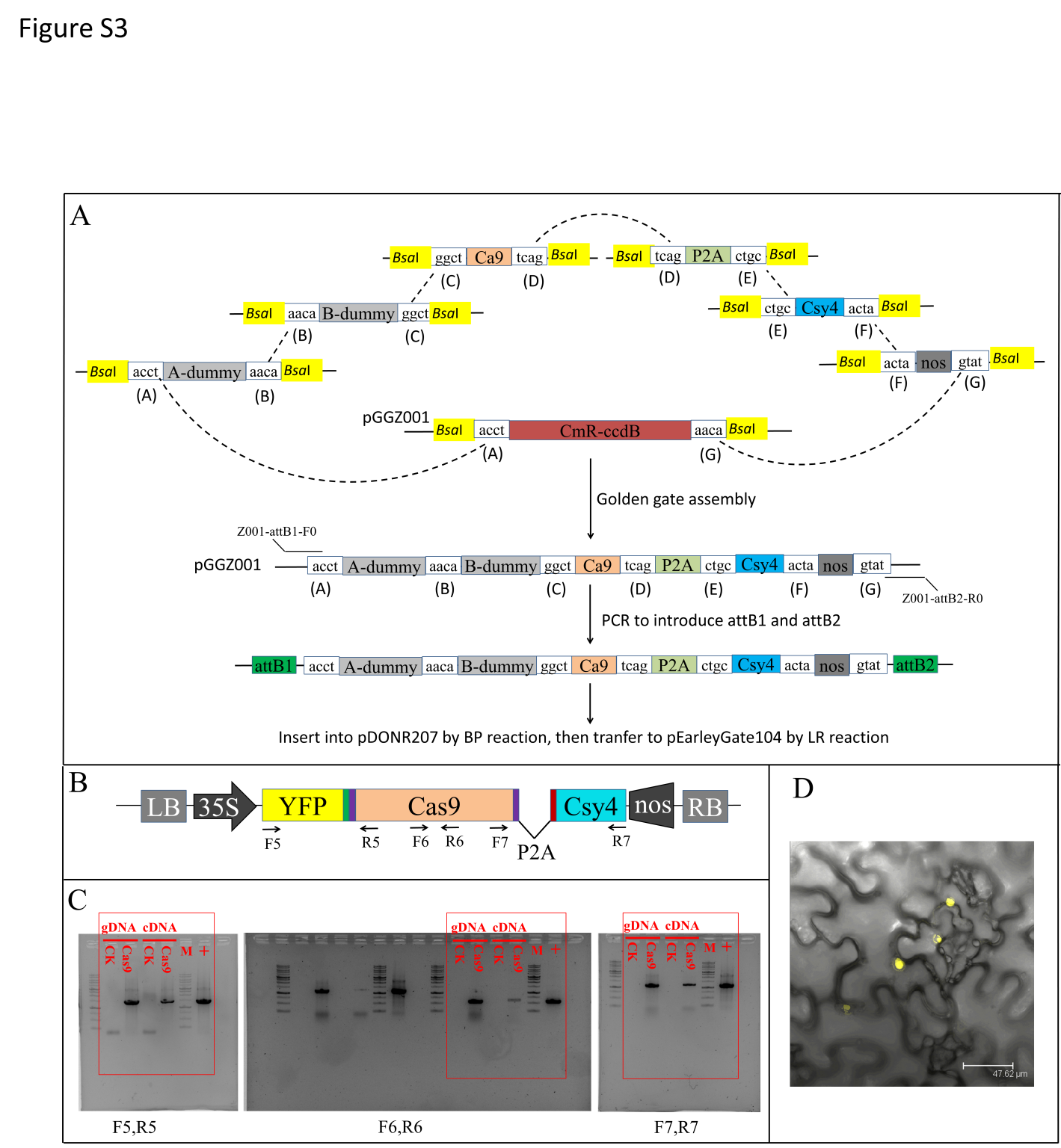


**Figure S6. Diagram and expression of Cas9-Csy4 construct.**

(A) Six module plasmids were assembled in order into pGGZ001 by Golden gate to create pGGZ001-Cas9-P2A-Csy4. The fragments were amplified from pGGZ001-Cas9-P2A-Csy4 with primers Z001-attB1-F0 and Z001-attB2-R0 to introduce attB1 and attB2 sequences. The PCR products were inserted into pDONR207 to produce p207-Cas9-P2A-Csy4 by BP reaction. Then the fragments were transferred to pEarleyGate104, producing binary plasmid pEG104-Cas9-P2A-Csy4.

(B) The positions of primers F5, R5, F6, R6, F7 and R7 to detect Cas9 and Csy4 expression.

(C) The expression of Cas9 and Csy4. Genomic DNA or RNA was extracted from *N. benthamiana* leaves infiltrated by empty *Agrobacterium* (CK) or pEG104-Cas9-P2A-Csy4 (Cas9). The cDNA was reverse transcribed from RNA. PCR was performed using indicated primer pairs. The positive control (+) was the PCR products with plasmid pEG104-Cas9-P2A-Csy4 as template.

(D) The expression of YFP-Cas9 fusion protein. The *N. benthamiana* leaves were infiltrated by pEG104-Cas9-P2A-Csy4 and detected by Confocal 2dpi.

ACCTccggcagcTccggcagcTccggcagcAACAgtATTCAGTCGACTggtaccaacaGGCTCCATGGATTACAAGGACCACGACATTGATTACAAGGATGATGATGACAAGATGGCTCCGAAGAAGAAGAGGAAGGTTGGCATCCACGGGGTGCCAGCTGCTGACAAGAAGTACTCGATCGGCCTCGACATTGGGACTAACTCTGTTGGCTGGGCCGTGATCACCGACGAGTACAAGGTGCCCTCAAAGAAGTTCAAGGTCCTGGGCAACACCGATCGGCATTCCATCAAGAAGAATCTCATTGGCGCTCTCCTGTTCGACAGCGGCGAGACGGCTGAGGCTACGCGGCTCAAGCGCACCGCCCGCAGGCGGTACACGCGCAGGAAGAATCGCATCTGCTACCTGCAGGAGATTTTCTCCAACGAGATGGCGAAGGTTGACGATTCTTTCTTCCACAGGCTGGAGGAGTCATTCCTCGTGGAGGAGGATAAGAAGCACGAGCGGCATCCAATCTTCGGCAACATTGTCGACGAGGTTGCCTACCACGAGAAGTACCCTACGATCTACCATCTGCGGAAGAAGCTCGTGGACTCCACAGATAAGGCGGACCTCCGCCTGATCTACCTCGCTCTGGCCCACATGATTAAGTTCAGGGGCCATTTCCTGATCGAGGGGGATCTCAACCCGGACAATAGCGATGTTGACAAGCTGTTCATCCAGCTCGTGCAGACGTACAACCAGCTCTTCGAGGAGAACCCCATTAATGCGTCAGGCGTCGACGCGAAGGCTATCCTGTCCGCTAGGCTCTCGAAGTCTCGGCGCCTCGAGAACCTGATCGCCCAGCTGCCGGGCGAGAAGAAGAACGGCCTGTTCGGGAATCTCATTGCGCTCAGCCTGGGGCTCACGCCCAACTTCAAGTCGAATTTCGATCTCGCTGAGGACGCCAAGCTGCAGCTCTCCAAGGACACATACGACGATGACCTGGATAACCTCCTGGCCCAGATCGGCGATCAGTACGCGGACCTGTTCCTCGCTGCCAAGAATCTGTCGGACGCCATCCTCCTGTCTGATATTCTCAGGGTGAACACCGAGATTACGAAGGCTCCGCTCTCAGCCTCCATGATCAAGCGCTACGACGAGCACCATCAGGATCTGACCCTCCTGAAGGCGCTGGTCAGGCAGCAGCTCCCCGAGAAGTACAAGGAGATCTTCTTCGATCAGTCGAAGAACGGCTACGCTGGGTACATTGACGGCGGGGCCTCTCAGGAGGAGTTCTACAAGTTCATCAAGCCGATTCTGGAGAAGATGGACGGCACGGAGGAGCTGCTGGTGAAGCTCAATCGCGAGGACCTCCTGAGGAAGCAGCGGACATTCGATAACGGCAGCATCCCACACCAGATTCATCTCGGGGAGCTGCACGCTATCCTGAGGAGGCAGGAGGACTTCTACCCTTTCCTCAAGGATAACCGCGAGAAGATCGAGAAGATTCTGACTTTCAGGATCCCGTACTACGTCGGCCCACTCGCTAGGGGCAACTCCCGCTTCGCTTGGATGACCCGCAAGTCAGAGGAGACGATCACGCCGTGGAACTTCGAGGAGGTGGTCGACAAGGGCGCTAGCGCTCAGTCGTTCATCGAGAGGATGACGAATTTCGACAAGAACCTGCCAAATGAGAAGGTGCTCCCTAAGCACTCGCTCCTGTACGAGTACTTCACAGTCTACAACGAGCTGACTAAGGTGAAGTATGTGACCGAGGGCATGAGGAAGCCGGCTTTCCTGTCTGGGGAGCAGAAGAAGGCCATCGTGGACCTCCTGTTCAAGACCAACCGGAAGGTCACGGTTAAGCAGCTCAAGGAGGACTACTTCAAGAAGATTGAGTGCTTCGATTCGGTCGAGATCTCTGGCGTTGAGGACCGCTTCAACGCCTCCCTGGGGACCTACCACGATCTCCTGAAGATCATTAAGGATAAGGACTTCCTGGACAACGAGGAGAATGAGGATATCCTCGAGGACATTGTGCTGACACTCACTCTGTTCGAGGACCGGGAGATGATCGAGGAGCGCCTGAAGACTTACGCCCATCTCTTCGATGACAAGGTCATGAAGCAGCTCAAGAGGAGGAGGTACACCGGCTGGGGGAGGCTGAGCAGGAAGCTCATCAACGGCATTCGGGACAAGCAGTCCGGGAAGACGATCCTCGACTTCCTGAAGAGCGATGGCTTCGCGAACCGCAATTTCATGCAGCTGATTCACGATGACAGCCTCACATTCAAGGAGGATATCCAGAAGGCTCAGGTGAGCGGCCAGGGGGACTCGCTGCACGAGCATATCGCGAACCTCGCTGGCTCGCCAGCTATCAAGAAGGGGATTCTGCAGACCGTGAAGGTTGTGGACGAGCTGGTGAAGGTCATGGGCAGGCACAAGCCTGAGAACATCGTCATTGAGATGGCCCGGGAGAATCAGACCACGCAGAAGGGCCAGAAGAACTCACGCGAGAGGATGAAGAGGATCGAGGAGGGCATTAAGGAGCTGGGGTCCCAGATCCTCAAGGAGCACCCGGTGGAGAACACGCAGCTGCAGAATGAGAAGCTCTACCTGTACTACCTCCAGAATGGCCGCGATATGTATGTGGACCAGGAGCTGGATATTAACAGGCTCAGCGATTACGACGTCGATCACATCGTTCCACAGTCATTCCTGAAGGATGACTCCATTGACAACAAGGTCCTCACCAGGTCGGACAAGAACCGGGGCAAGTCTGATAATGTTCCTTCAGAGGAGGTCGTTAAGAAGATGAAGAACTACTGGCGCCAGCTCCTGAATGCCAAGCTGATCACGCAGCGGAAGTTCGATAACCTCACAAAGGCTGAGAGGGGCGGGCTCTCTGAGCTGGACAAGGCGGGCTtcatcaagaggcagctggtcgagacacggcagatcaCTAAGCACGTTGCGCAGATTCTCGACTCACGGATGAACACTAAGTACGATGAGAATGACAAGCTGATCCGCGAGGTGAAGGTCATCACCCTGAAGTCAAAGCTCGTCTCCGACTTCAGGAAGGATTTCCAGTTCTACAAGGTTCGGGAGATCAACAATTACCACCATGCCCATGACGCGTACCTGAACGCGGTGGTCGGCACAGCTCTGATCAAGAAGTACCCAAAGCTCGAGAGCGAGTTCGTGTACGGGGACTACAAGGTTTACGATGTGAGGAAGATGATCGCCAAGTCGGAGCAGGAGATTGGCAAGGCTACCGCCAAGTACTTCTTCTACTCTAACATTATGAATTTCTTCAAGACAGAGATCACTCTGGCCAATGGCGAGATCCGGAAGCGCCCCCTCATCGAGACGAACGGCGAGACGGGGGAGATCGTGTGGGACAAGGGCAGGGATTTCGCGACCGTCAGGAAGGTTCTCTCCATGCCACAAGTGAATATCGTCAAGAAGACAGAGGTCCAGACTGGCGGGTTCTCTAAGGAGTCAATTCTGCCTAAGCGGAACAGCGACAAGCTCATCGCCCGCAAGAAGGACTGGGATCCGAAGAAGTACGGCGGGTTCGACAGCCCCACTGTGGCCTACTCGGTCCTGGTTGTGGCGAAGGTTGAGAAGGGCAAGTCCAAGAAGCTCAAGAGCGTGAAGGAGCTGCTGGGGATCACGATTATGGAGCGCTCCAGCTTCGAGAAGAACCCGATCGATTTCCTGGAGGCGAAGGGCTACAAGGAGGTGAAGAAGGACCTGATCATTAAGCTCCCCAAGTACTCACTCTTCGAGCTGGAGAACGGCAGGAAGCGGATGCTGGCTTCCGCTGGCGAGCTGCAGAAGGGGAACGAGCTGGCTCTGCCGTCCAAGTATGTGAACTTCCTCTACCTGGCCTCCCACTACGAGAAGCTCAAGGGCAGCCCCGAGGACAACGAGCAGAAGCAGCTGTTCGTCGAGCAGCACAAGCATTACCTCGACGAGATCATTGAGCAGATTTCCGAGTTCTCCAAGCGCGTGATCCTGGCCGACGCGAATCTGGATAAGGTCCTCTCCGCGTACAACAAGCACCGCGACAAGCCAATCAGGGAGCAGGCTGAGAATATCATTCATCTCTTCACCCTGACGAACCTCGGCGCCCCTGCTGCTTTCAAGTACTTCGACACAACTATCGATCGCAAGAGGTACACAAGCACTAAGGAGGTCCTGGACGCGACCCTCATCCACCAGTCGATTACCGGCCTCTACGAGACGCGCATCGACCTGTCTCAGCTCGGGGGCGACTCAGGTCCTAAGAAGAAGAGGAAGGTTTCAGgtGGCAGTGGAGAGGGCAGAGGAAGTCTGCTAACATGCGGTGACGTCGAGGAGAATCCTGGCCCAATGCTGCCCATGGAACAAAAATTAATCTCAGAAGAAGACTTGAACGGTGAACAAAAGCTAATCTCCGAGGAAGACTTGGGTAGTGACCACTATCTGGACATCAGACTGAGGCCCGATCCTGAGTTCCCTCCCGCCCAGCTGATGAGCGTGCTGTTTGGCAAGCTGCATCAGGCTCTGGTCGCCCAAGGCGGAGACAGAATCGGCGTGTCCTTCCCCGACCTGGACGAGTCCCGGAGTCGCCTGGGCGAGCGGCTGAGAATCCACGCCAGCGCAGACGATCTGCGCGCCCTGCTGGCCCGGCCTTGGCTGGAGGGCCTGCGGGATCATCTGCAGTTTGGCGAGCCCGCCGTGGTGCCACACCCAACACCCTACCGCCAGGTGAGCCGCGTGCAGGCCAAGTCAAATCCCGAGAGACTGCGGCGGAGGCTGATGAGGCGACATGATCTGAGCGAGGAGGAGGCCAGAAAGAGAATCCCCGACACAGTGGCCAGAGCCCTGGATCTGCCATTTGTGACCCTGCGGAGCCAGAGCACTGGCCAGCATTTCAGACTGTTCATCAGACACGGGCCCCTGCAGGTGACAGCCGAGGAGGGCGGATTTACATGCTATGGCCTGTCTAAAGGCGGCTTCGTGCCCTGGTTCTGAACTATAAGTAGGTGACTGCAGATCGTTCAAACATTTGGCAATAAAGTTTCTTAAGATTGAATCCTGTTGCCGGTCTTGCGATGATTATCATATAATTTCTGTTGAATTACGTTAAGCATGTAATAATTAACATGTAATGCATGACGTTATTTATGAGATGGGTTTTTATGATTAGAGTCCCGCAATTATACATTTAATACGCGATAGAAAACAAAATATAGCGCGCAAACTAGGATAAATTATCGCGCGCGGTGTCATCTATGTTACTAGATCCGATGATAAGCTGTCAAACATGAGAATTCGTAT

**Figure S7. *Bsa*I cloning site sequence in plasmid pGGZ001-Cas9-P2A-Csy4.**

Cas9 coding sequence underlined, P2A sequence highlighted in yellow, Csy4 sequence double-underlined, nos terminator sequence wavy underlined. The start and stop code of the open reading frame colored in red, Flag tag colored in green, NLS colored in purple, Myc tag colored in pink. *Bsa*I restriction overhangs created during Golden gate assembly written in box.
