# Supplementary material for: Development of a Csy4-processed guide RNA delivery system with soybean-infecting virus ALSV for genome editing
Source: BMC Plant Biol. 2021 Sep 13;21:419. doi: 10.1186/s12870-021-03138-8 (PMC8436479; doi:10.1186/s12870-021-03138-8)
Supplement: Supplementary file 2 — Additional File 2: Original uncropped gel pictures Figure S1. Uncropped full-length gels of Figure 2C. Figure S2. Uncropped full-length gels of Figure 3C. Figure S3. Uncropped full-length gels of Figure 4C. Figure S4. Uncropped full-length gels of Figure 4D. [file 12870_2021_3138_MOESM2_ESM.docx]

**Additional file 2:**

**Original uncropped gel pictures**

**
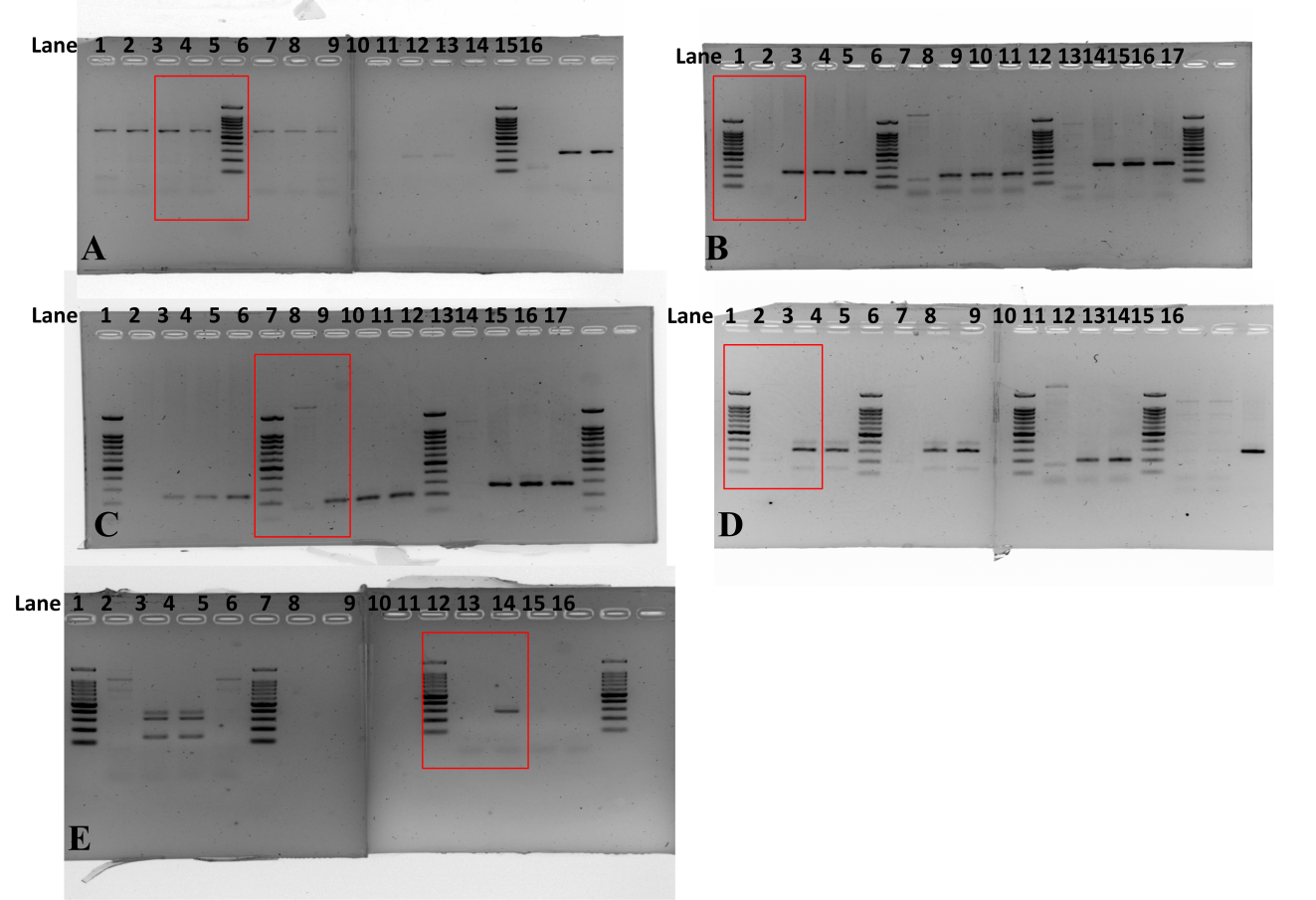
**

**Figure S1.** Uncropped full-length gels of Figure 2C. A-E Gels corresponding to Figure 2C gels from top to bottom accordingly. A. Cropped from lane 3 to lane 5 (included), and dragged the ladder side to the left. B.Cropped from lane 1 to lane 3 (included). C. Cropped from lane 6 to lane 8 (included). D.Cropped from lane 1 to lane 3 (included). E.Cropped from lane 10 to lane 12 (included).

**
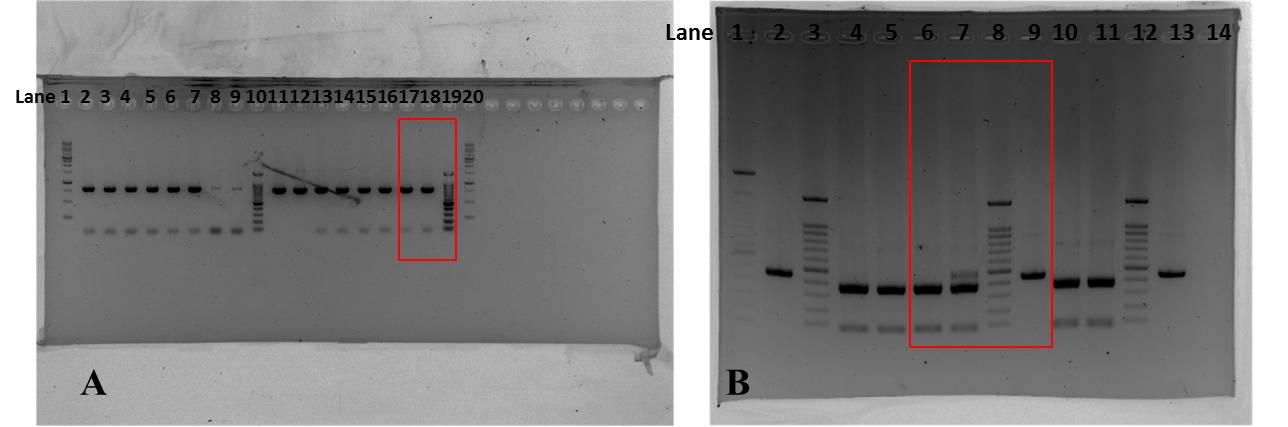
**

**Figure S2.** Uncropped full-length gels of Figure 3C. A,B Gels corresponding to figure 3C gels from top to bottom. A. Cropped from lane 17 to lane 19 (included), and dragged the ladder side to the left. B.Cropped from lane 6 to lane 9 (included). and dragged right side to the left.

**
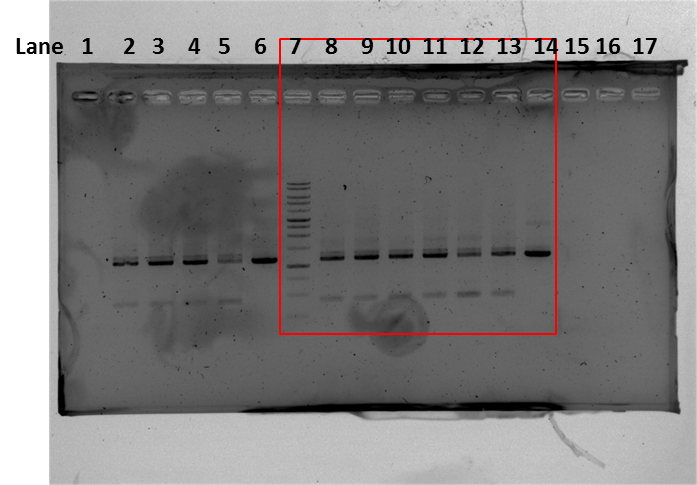
**

**Figure S3.** Uncropped full-length gels of Figure 4C. Cropped from lane 7 to lane 14 (included).

**
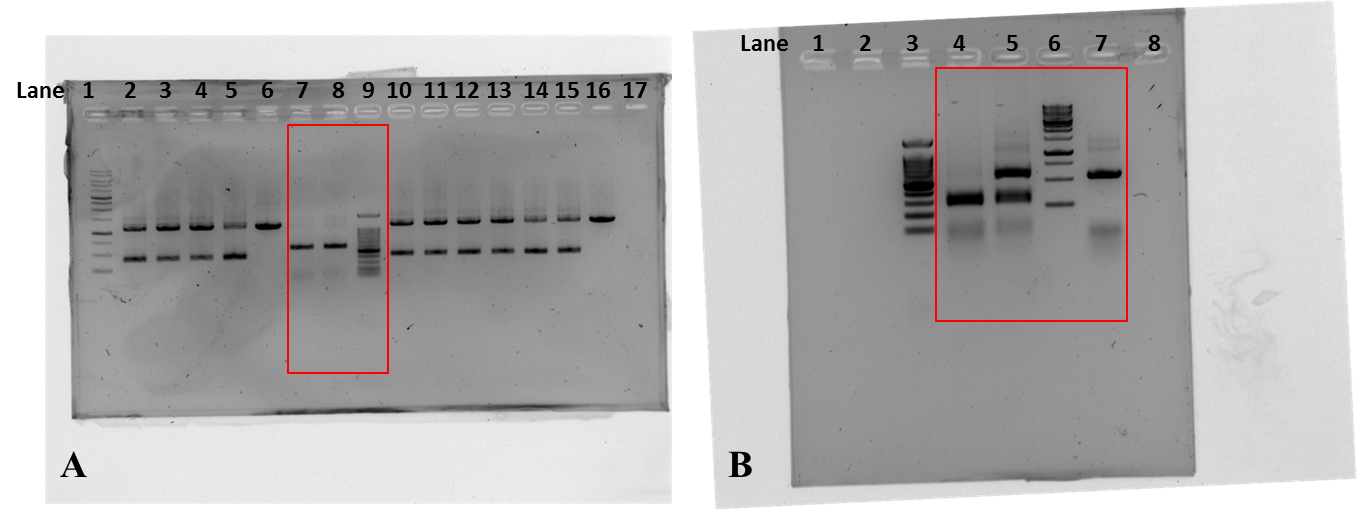
**

**Figure S4.** Uncropped full-length gels of Figure 4D. A,B Gels corresponding to figure 4D gels from left to right. A. Cropped from lane 7 to lane 9 (included), and dragged the ladder side to the left. B.Cropped from lane 4 to lane 7 (included) and dragged right side to the left.
