# Supplementary material for: Development of a Csy4-processed guide RNA delivery system with soybean-infecting virus ALSV for genome editing
Source: BMC Plant Biol. 2021 Sep 13;21:419. doi: 10.1186/s12870-021-03138-8 (PMC8436479; doi:10.1186/s12870-021-03138-8)
Supplement: Supplementary file 3 — Additional File 3. Sanger sequencing raw data The Sanger sequencing raw data of genomic editing, including PDS gene, EPSPS gene and GW2 gene. [file 12870_2021_3138_MOESM3_ESM.docx]

Additional file 3

Sanger sequencing data

EPSPS target

>M1S_1_2R_PREMIX Sample_Name=M1S_1_2R_PREMIX Chromat_id=7120862 Read_id=7059583 Version=1 Length=506

GGTGCCAGCTGCGCTGGATGGCAATAATGATTTTATTTTGACTGATAGTGACCTGTTCGTTGCAACAAATTGATAAGCAATGCTTTCTTATAATGCCAACTTTGTACAAGAAAGCTGGGTCCCCAATACTTGTATGGCATACCACAATCAACATCTGCACCAAGCTGCTTCAGACCAGCAACCAAATCACCAATTGGCCTCTCTCTCATTCGAGGCACTCCATCGAGTACATAGCTGAAATATTTTGAACATCGAGATGGTCTATTTAGAGACTCAAGCCAGTAATCATGATAGAAGATCAAATTATAACAGAAGAAAATGATGAGATTGAAGTAGATCATGAGGAAGACCCTCAGTTGCATTCCTCAGAATAGGACGCTTCATTTTCACAGAAGATTACAAAAATTCAAATATTCAAAATCTGGATTACAGCTCCTAATTACATAACCGCAAAAAAAAAAATACAAATAAATGAATAAACTTCACCTCAAATAGAGCCAGCAAGA

>M2S_11_2R_PREMIX Sample_Name=M2S_11_2R_PREMIX Chromat_id=7120870 Read_id=7059595 Version=1 Length=1075

CGGGGACAGCCTGCGCCTGGATGGCAATAATGATTTTATTTTGACTGATAGTGACCTGTTCGTTGCAACAAATTGATAAGCAATGCTTTCTTATAATGCCAACTTTGTACAAGAAAGCTGGGTCCCCAATACTTGTATGGCATACCACAATCAACATCTGCACCAAGCTGCTTCAGACCAGCAACCAAATCACCAATTGGCCTCTCTCTCATTCGAGGCACTCCATCGAGTACATAGCTGAAATATTTTGAACATCGAGATGGTCTATTTAGAGACTCAAGCCAGTAATCATGATAGAAGATCAAATTATAACAGAAGAAAATGATGAGATTGAAGTAGATCATGAGGAAGACCCTCAGTTGCATTCCTCAGAAGATTCAAATTATAATAGGACGCTTCATTTTCACAGAAGATTACAAAAATTCAAATATTCAAAATCTGGATTACAGCTCCTAATTACATAACCGCAAAAAAAAAATACAAATAAATGAATAAACTTCACCTCAGATAAGGCTTGTAACGATTTTCTCATAAAAAGTTCTTCGAGTAATTTTCTAATCTCATAATGAACAAACCATATATTGCTTTCCCCTTGCATTCCCACCAGCTGCTACAACAGCTGCTGTGGCAGGCCGTCCTTTACTAGCAGCATTACCTAGGTATATATTGATTCCTTCGTAAGAGTCTCCACAGGCGAAAACAACCCCACCCGCCCTTTACAGTTGCTCCGCTCTGGCAGTTAGCCACCTTCCACAGCCAGGCCGAGGGGACTTAAGCCGCATGCATAGAATGAACATCCTCGCCATCCATCTGCTGTTACAAAAGTTCTTATCTATTTAGAAAGACCAGACTGCTTATTTTAGGTTCTACAAGTCCATAATATATGCTAATGAGAGGTGCACAACCTATCGAATGCAACGTGGCTCGTACCTTGGAACTACCACTTCTGGCCTTGGGCCCTTGCGGTCATCTCTCAGGGTCGATTAGTGCCCCCAAGACTGCATTAACGGGGGAACATTGCAGGAGGTCTGATGTCATTTGGTCTATTAGATCCTTAATTTGTCATACTTGTGCT

>M2S_13_2R_PREMIX Sample_Name=M2S_13_2R_PREMIX Chromat_id=7120871 Read_id=7059587 Version=1 Length=1078

GGGTGCGAGCTGCGCTGGATGGCAATAATGATTTTATTTTGACTGATAGTGACCTGTTCGTTGCAACAAATTGATAAGCAATGCTTTCTTATAATGCCAACTTTGTACAAGAAAGCTGGGTCCCCAATACTTGTATGGCATACCACAATCAACATCTGCACCAAGCTGCTTCAGACCAGCAACCAAATCACCAATTGGCCTCTCTCTCATTCGAGGCACTCCATCGAGTACATAGCTGAAATATTTTGAACATCGAGATGGTCTATTTAGAGACTCAAGCCAGTAATCATGATAGAAGATCAAATTATAACAGAAGAAAATGATGAGATTGAAGTAGATCATGAGGAAGACCCTCAGTTGCATTCATCATAATAGGACGCTTCATTTTCACAGAAGATTACAAAAATTCAAATATTCAAAATCTGGATTACAGCTCCTAATTACATAACCGCAAAAAAAAAATACAAATAAATGAATAAACTTCACCTCAGATAAGCAGCAAGAAAGCACATGTTTTGTCCAGTGAGGTTATCCCCCAAAAATTCTACTATCCAAATGGATTAACCAAATAAAAAACAAAACCTTGCATTTTCTCTGGCGGCACCAACAGGCGCTGTGCGAGGGACCGGCTGGTGTCACCAGGACTTCCCCAGGGGAATAAATTGGTTTCCTCTTTAAGATTCTCTCATGAGGGGGGAAAAGAAATCCCCCCCCCCAGCCCTCCCCCCATTCCCTCCCGCTGGGTTTGTTTTGTTTTCTCTCCCCATGGCCAGTCCTAAGGGTCCCTTAAAGCCCCCCAGCCCCGGAAAGGTATATTCCCCCCCCCCTAACAACACAAGGTCGTCCTCATCCAAGTGGTCCCCCCTTATTCTGTAAAAAAAACAGGAAAATTAAATTTTATGTGCCCACACACAGCCCTCCTATCTCTATGTTCTACTTTTTAAGAGGTTGTTCGACAACATATCCGAGATTAGGATAAAATGTTGTGCTATCCATAAAACACTTGGCTAGAAAACCCCCCCCCCTACCTCTTCGCGGTGGTTCGGTGGTGGCCGCTCCTTTGGTGGGAGTTGTAGCT

>M2S_17_2R_PREMIX Sample_Name=M2S_17_2R_PREMIX Chromat_id=7120874 Read_id=7059603 Version=1 Length=1054

GGGTACGCGCTGCAGCTGGATGGCAATAATGATTTTATTTTGACTGATAGTGACCTGTTCGTTGCAACAAATTGATAAGCAATGCTTTCTTATAATGCCAACTTTGTACAAGAAAGCTGGGTCCCCAATACTTGTATGGCATACCACAATCAACATCTGCACCAAGCTGCTTCAGACCAGCAACCAAATCACCAATTGGCCTCTCTCTCATTCGAGGCACTCCATCGAGTACATAGCTGAAATATTTTGAACATCGAGATGGTCTATTTAGAGACTCAAGCCAGTAATCATGATAGAAGATCAAATTATAACAGAAGAAAATGATGAGATTGAAGTAGATCATGAGGAGGACCCTCAGTTGCATTCCTCAGAAGATTCAAATTATAATAGGACGCTTCATTTTCACAGAAGATTACAAAAATTCAAATATTCAAAATCTGGATTACAGCTCCTAATTACATAACCGCAAAAAAAAAAATACAAATAAATGAATAAACTTCACCTCAGAAGGGCAGCAAGAAAATTCTGATACGTCATGGTAATTCACATATCAAAAAGCATAAAGAAAATACAAAAAAGACATTGCATTTGGACCTGCCCCCATGACCCCCCGACGTCCAGCGGCCCATTGCAGCACTGCGCTTACCGCATTAAAAAGTGATTATATTGTTTAGATTCTTCAGGAGCGGCAAACAGTGGCCAAACAAGCCTTCCAACAATTGCCTCGCCATTGGCGTGCTTGCTTCGTTTGGCCGGCTGTGCGAGGGGTCCTCCAAGGGGCCCGGAATGGCACCGAGTATCTTCTGCAATACAACACGGCTGTACACTCACAGTTGCTTCCCACTAGTTGGACGCCCTCCAGTACCTTAAACTCGAGTGACCCACAAATCTAATGCTCTAATTGACATTTATCACAGTTCGTGCAAAATTAAGTAAGATGTGAAAGAAGAAAGCCGAAACTTGAAATACCAACCCATCTCCAGAGCTTGGGGAAGGCCACTGGGAGTTCTATATGCATTTCATGAGGAATTCTTAGGAAGATTTTTGAGCTCTT

>M3S_3_2R_PREMIX Sample_Name=M3S_3_2R_PREMIX Chromat_id=7120887 Read_id=7059618 Version=1 Length=993

GGGGGAAAGCTGCGCTGGATGGCAATAATGATTTTATTTTGACTGATAGTGACCTGTTCGTTGCAACAAATTGATAAGCAATGCTTTCTTATAATGCCAACTTTGTACAAGAAAGCTGGGTCCCCAATACTTGTATGGCATACCACAATCAACATCTGCACCAAGCTGCTTCAGACCAGCAACCAAATCACCAATTGGCCTCTCTCTCATTCGAGGCACTCCATCGAGTACATAGCTGAAATATTTTGAACATCGAGATGGTCTATTTAGAGACTCAAGCCAGTAATCATGATAGAAGATCAAATTATAACAGAAGAAAATGATGAGATTGAAGTAGATCATGAGGAAGACCCTCAGTTGCATTCCTCAGTAATAGGACGCTTCATTTTCACAGAAGATTACAAAAATTCAAATATTCAAAATCTGGATTACAGCTCCTAATTACATAACCGCAAAAAAAAAAATACAAATAAATGAATAAACTTCACCTCAGATAGGGCAGCAAGAAAGGTGTTTTCTCTCCCTGATGACATTCTACGGTATATTATTGCTCTTTGTAGGTGAAGAAAATAAATGAAATTCGTTTTTGGTTTTTCATCCTGCCTCCCCTTGGCTGGTGATCAAGAGAAGCACCGGTCTGCCCCACAACCCAATGGATTTAATTGTTTAAATGCTGTAAAAAAGAACCAAAAATGGGAGCCTCGCCCTCCCTCAGACCTCTCCACTCTTGGCTGTTTTCGGTTGGGTTAATGTCCATCCGACCAAAATGTCGTTTCAACCCAATCCTTTACAGTTGACGAATCCATGGTCTCGAAATACTCGCTCCGATCTTCCAATCCAGGTGGCCCTCCCGTTCCCGTTGTGCTACCCATCATATAATAAAAATTGAAAGTCCCCTTCAATTTCCACGGCGCCCAACTCGCTCATGCCCACGCTTGTCCCTTGCTTCAGAAGGAGGGGTAAAAGTAAGAAACTTGCCAATGGTGGTCGTCA

>M3S_5_2R_PREMIX Sample_Name=M3S_5_2R_PREMIX Chromat_id=7120905 Read_id=7059559 Version=1 Length=644

GGTACAAGCCTGCGCTGGATGGCAATAATGATTTTATTTTGACTGATAGTGACCTGTTCGTTGCAACAAATTGATAAGCAATGCTTTCTTATAATGCCAACTTTGTACAAGAAAGCTGGGTCCCCAATACTTGTATGGCATACCACAATCAACATCTGCACCAAGCTGCTTCAGACCAGCAACCAAATCACCAATTGGCCTCTCTCTCATTCGAGGCACTCCATCGAGTACATAGCTGAAATATTTTGAACATCGAGATGGTCTATTTAGAGACTCAAGCCAGTAATCATGATAGAAGATCAAATTATAACAGAAGAAAATGATGAGATTGAAGTAGATCATGAGGAAGACGCTTCATTTTCACAGAAGATTACAAAAATTCAAATATTCAAAATCTGGATTACAGCTCCTAATTACATAACCGCAAAAAAAAAAATACAAATAAATGAATAAACTTCACCTCAGATAAGCTCTTAATGAAAACCTTCTCTTTATTGACACAGGCGGGAGGAGGAATGTGAACTTATCTTCTGGTCTGATAATACCCCACGACATTCAATTCTCATATTGAAATGACAAAACAAAAAACAAACCTGGCATTGTAACTTCCCCCCCCAGCCAGCCTGCGGTGCAGTCCGCATTTG

GW2 target

>S6_1_2R_PREMIX Sample_Name=S6_1_2R_PREMIX Chromat_id=7158988 Read_id=7096213 Version=1 Length=759

NNNNNNCNNNGCTGGNNGGNAATAATGATTTNATTTTGACTGATAGTGACCTGTTCGTTGCAACAAATTGATAAGCAATGCTTTCTTATAATGCCAACTTTGTACAAGAAAGCTGGGTCCCCAATACTTGTATGGCATACCAAATTTGCAGTTTGAAAGAAGTGTGCACAGTTGAATTGACATATTTATTTATTTTTCTAGAAAGCTAGAGTAACAGGATAAGTAGAGAGAAACAAATATTGAAGGCATGCAATAGAAATCATACCAGAAAGCAAATCGGGCACTCTTCACGATCGTAGGTGGTTTCTTCATCTCCGGGATAGCAAGGTGCTAACTTTGATTCTAGTATTAGCTTCCTCAGCTTCTTGTGATCCACATCTTTGTGATTATACAACCCTTGCGCCTCGTATATTTCTCATCTACCACTTGTCTCCTCCTCCCAAGTTTATTACCCATCCAACAAAACTTAATTCTTCTCGTCTGCCGACTATGTTCCTACTCTTAAACAAAATAAACTACCACAACAACAACAAAAATAACAAGCCTTCCTCTACAAAAACCACTCTCTCAACCACATCACGAAATCATCTTCACTGAACATAAGATTGTACTCAACAAACAACAATCTAACAGCCAAACCCCCCATCTACAACACAATCAACAGAANNGGGGGNACCNNNTTAAAA

>S6_1_19_2R_PREMIX Sample_Name=S6_1_2R_PREMIX Chromat_id=7231334 Read_id=7158988 Version=1 Length=697

NNNNNNNNNNTGCGCTGGNTGGCAATAATGATTTTATTTTGACTGATAGTGACCTGTTCGTTGCAACAAATTGATAAGCAATGCTTTCTTATAATGCCAACTTTGTACAAGAAAGCTGGGTCCCCAATACTTGTATGGCATACCAAATTTGCAGTTTGAAATAAGTGTGAACAGTTGAATTGACATATTTGCTTCTTTTTTCTTTTTCTTTTTTTTTTCTAAAAAGCTAGAGTAACAGGATAAGTAGAGAGAAACAAATATTAAAGGCATGCGATAGAAACCATACCAGAAAGCAAATCGGGCACTCTTCACGATCGTATGCGGTTTCTTCATCTCCGGGATAGCAAGGTGCTAACTTCGATTCTAGTATTAGCTTCCTCAGCTTCTTGTGATCCACATCTTTATGATTATACAACCCTTGCGGGCCNNNTNNATCTCTCACNANCCACTTGCCCCCCCNTCCCAAATTAATTACCCANNAAAAANTTCTAATTTTTCTCGCCCGCNNNCTACGTACCGAACTCTNNANCAAAATAAACCAACCCACCACCACCACCACCAACAAANNTAACAAACCTTNNANAAAAAAANCCACTCACTCACCCACATCACAAAATCATCTTCANNNNAAACCANNTNACGTTAACGCCACACTNANCAANCCAACNAATCANNNAGGNNNNNAAACAATTTNNAA

>S6_2_2R_PREMIX Sample_Name=S6_2_2R_PREMIX Chromat_id=7158989 Read_id=7096208 Version=1 Length=686

GAGAGCTGCGCTGGGATGGCAATAATGATTTTATTTTGACTGATAGTGACCTGTTCGTTGCAACAAATTGATAAGCAATGCTTTCTTATAATGCCAACTTTGTACAAGAAAGCTGGGTCCCCAATACTTGTATGGCATACCAAATTTGCAGTTTGAAAGAAGTGTGCACAGTTGAATTGACATATTTATTTATTTTTCTAGAAAGCTAGAGTAACAGGATAAGTAGAGAGAAACAAATATTGAAGGCATGCAATAGAAATCATACCAGAAAGCAAATCGGGCACTCTTCACGATCGTAGGTGGTTTCTTCATCTCCGGGATAGCAAGGTGCTAACTTTGATTCTAGTATTAGCTTCCTCAGCTTCTTGTGATCCACATCTTTGTGATTATACAACCCTTGCGCCTCGTATATTTCTCATCTACCACTTGTCTCCTCCTCCCAAGTTTATTACCCATCCAACAAAACTTAATTCTTCTCGTCTGCCGACTATGTTCCTACTCTTAAACAAAATAAACTACCACAACAACAACAAAAATAACAAGCCTTCCTCTACAAAAACCACTCTCTCAACCACATCACGAAATCATCTTCACTGAACATAAGATTGTACTCAACAAACAACAATCTAACAGCCAAACCCCCCATCTACAACACAATCAACAGAAGGATGAAAAACTCGAGTAAA

>S6_2_19_2R_PREMIX Sample_Name=S6_2_2R_PREMIX Chromat_id=7231335 Read_id=7158985 Version=1 Length=725

NNNNNNNNNCTGCGCTGGNNGGCAATAATGATTTTATTTTGACTGATAGTGACCTGTTCGTTGCAACAAATTGATAAGCAATGCTTTCTTATAATGCCAACTTTGTACAAGAAAGCTGGGTCCCCAATACTTGTATGGCATACCAAATTTGCAGTTTGAAATAAGTGTGAACAGTTGAATTGACATATTTGCTTCTTTTTTCTTTTTCTTTTTTTTTTCTAAAAAGCTAGAGTAACAGGATAAGTAGAGAGAAACAAATATTAAAGGCATGCGATAGAAACCATACCAGAAAGCAAATCGGGCACTCTTCACGATCGTATGCGGTTTCTTCATCTCCGGGATAGCAAGGTGCTAACTTCGATTCTAGTATTAGCTTCCTCAGCTTCTTGTGATCCACATCTTTATGATTATACAACCCTTGCGGGCCTCGTATATTTCTCATCTACCACTTGTCTCCTCCTCCCAAGTTTATTACCCATCCAAAAATTCCAGTTTTTCTCGTCCGCCGACTATGTTCCGAACTCTTCAACAAAATAAACCAACACAACAACAACAACAACAACAAAAGTAACAAACCTTCCACTACAAAAACCACTCACTCAACCACATCACAAAATCATCTTCACTCAAAACCAAATTACGTTAACGCCACACTCAACAAACCAACACAATCAACAGAGTGNNNNNNNCCCTTTTTTANNAANNNNNNNNNNNNNNNNNAAAAA

>S6_3_2R_PREMIX Sample_Name=S6_3_2R_PREMIX Chromat_id=7158990 Read_id=7096195 Version=1 Length=696

GCGAAGGCTGAGCTGGATGGCAATAATGATTTTATTTTGACTGATAGTGACCTGTTCGTTGCAACAAATTGATAAGCAATGCTTTCTTATAATGCCAACTTTGTACAAGAAAGCTGGGTCCCCAATACTTGTATGGCATACCAAATTTGCAGTTTGAAATAAGTGTGAACAGTTGAATTGACATATTTGCTTCTTTTTTCTTTTTCTTTTTTTTTTCTAAAAAGCTAGAGTAACAGGATAAGTAGAGAGAAACAAATATTAAAGGCATGCGATAGAAACCATACCAGAAAGCAAATCGGGCACTCTTCACGATCGTATGCGGTTTCTTCATCTCCGGGATAGCAAGGTGCTAACTTCGATTCTAGTATTAGCTTCCTCAGCTTCTTGTGATCCACATCTTTATGATTATACAACCCTTGCGGGCCTCGTATATTTCTCATCTACCACTTGTCTCCTCCTCCCAAGTTTATTACCCATCCAAAAATTCCAATTTTTCTCGTCCGCCGACTATGTTCCGAACTCTTCAACAAAATAAACCAACACAACAACAACAACAACAACAAAAGTAACAAACCTTCCACTACAAAAACCACTCACTCAACCACATCACAAAATCATCTTCACTCAAAACCAAATTACGTTAACGCCACACTCAACAAACCAACACAATCAACAGAAGGAAAAAAACCCTTTTTA

>S6_3_19_2R_PREMIX Sample_Name=S6_3_2R_PREMIX Chromat_id=7231336 Read_id=7158979 Version=1 Length=684

NNNNNNNNCNNNNCTGGATGGCAATAATGNTTTTATTTTGACTGATAGTGACCTGTTCGTTGCAACAAATTGATAAGCAATGCTTTCTTATAATGCCAACTTTGTACAAGAAAGCTGGGTCCCCAATACTTGTATGGCATACCAAATTTGCAGTTTGAAATAAGTGTGAACAGTTGAATTGACATATTTGCTTCTTTTTTCTTTTTCTTTTTTTTTTCTAAAAAGCTAGAGTAACAGGATAAGTAGAGAGAAACAAATATTAAAGGCATGCGATAGAAACCATACCAGAAAGCAAATCGGGCACTCTTCACGATCGTATGCGGTTTCTTCATCTCCGGGATAGCAAGGTGCTAACTTCGATTCTAGTATTAGCTTCCTCAGCTTCTTGTGATCCACATCTTTATGATTATACAACCCTTGCNNNATTTCTCATCTACCACTTGTCTCCTCCTCCCNNNTTTATTACCCNTCNAAAAATTNNAATTTTTCTCGCCCGCCGACCATGTACNNAACTCATCCACAAAATAAACCAACCCAACAACCACCACCACCACCAACGTAACAAACCTTCCANNANAAAANCCACTCACTCAACCACATCAAAANNTCATCTTCACTCANANCCAAATTACTTACACGNNNACTCAACAAACCAACCANNCAANNNNNNGGGNNNNNNTAAAA

>S6_4_2R_PREMIX Sample_Name=S6_4_2R_PREMIX Chromat_id=7158995 Read_id=7096196 Version=1 Length=762

NNNNNNNNNNNNNTGGANGGCAATAATGATTTNATTTTGACTGATAGTGACCTGTTCGTTGCAACAAATTGATAAGCAATGCTTTCTTATAATGCCAACTTTGTACAAGAAAGCTGGGTCCCCAATACTTGTATGGCATACCAAATTTGCAGTTTGAAAGAAGTGTGCACAGTTGAATTGACATATTTATTTATTTTTCTAGAAAGCTAGAGTAACAGGATAAGTAGAGAGAAACAAATATTGAAGGCATGCAATAGAAATCATACCAGAAAGCAAATCGGGCACTCTTCACGATCGTAGGTGGTTTCTTCATCTCCGGGATAGCAAGGTGCTAACTTTGATTCTAGTATTAGCTTCCTCAGCTTCTTGTGATCCACATCTTTGTGATTATACAACCCTTGCGCCTCGTATATTTCTCATCTACCACTTGTCTCCTCCTCCCAAGTTTATTACCCATCCAACAAAACTTAATTCTTCTCGTCTGCCGACTATGTTCCTACTCTTAAACAAAATAAACTACCACAACAACAACAAAAATAACAAGCCTTCCTCTACAAAAACCACTCTCTCAACCACATCACGAAATCATCTTCACTGAACATAAGATTGTACTCAACAAACAACAATCTAACAGCCAAACCCCCCATCTACAACACAATCAACAGANNNNNNNANNCCCNTTTAAANNNNNNNNNNNNNNNNNNNNNNNNNNNNNNNNNNNNNNNNNNNNNNNNNNNNNNNNNNNNNNNNNNNNNNNNNNNN

>S6_4_19_2R_PREMIX Sample_Name=S6_4_2R_PREMIX Chromat_id=7231337 Read_id=7158983 Version=1 Length=696

NNNNNNNNNNTNCGCTGGNNGGNAATAATGATTTTATTTTGACTGATAGTGACCTGTTCGTTGCAACAAATTGATAAGCAATGCTTTCTTATAATGCCAACTTTGTACAAGAAAGCTGGGTCCCCAATACTTGTATGGCATACCAAATTTGCAGTTTGAAATAAGTGTGAACAGTTGAATTGACATATTTGCTTCTTTTTTCTTTTTCTTTTTTTTTTCTAAAAAGCTAGAGTAACAGGATAAGTAGAGAGAAACAAATATTAAAGGCATGCGATAGAAACCATACCAGAAAGCAAATCGGGCACTCTTCACGATCGTATGCGGTTTCTTCATCTCCGGGATAGCAAGGTGCTAACTTCGATTCTAGTATTAGCTTCCTCAGCTTCTTGTGATCCACATCTTTATGATTATACAACCCTTGCGCCTCGTATATTTCTCATCTACCACTTGTCTCCTCCTCCCAAGTTTATTACCCATCCAAAAATTCCAATTTTTCTCGTCCGCCGACTATGTTCCGAACTCTTCAACAAAATAAACCAACACAACAACAACAACAACAACAAAAGTAACAAACCTTCCACTACAAAAACCACTCACTCAACCACATCACAAAATCATCTTCACTCAAAACCAAATTACGTTAACGCCACACTCAACAAACCAACACAATCAACAGAANNGGGAAANNNNTTTTNA

>S6_5_2R_PREMIX Sample_Name=S6_5_2R_PREMIX Chromat_id=7158994 Read_id=7096190 Version=1 Length=782

NNNNNNNNNNNCTGGATGGCAATAATGATTTNATTTTGACTGATAGTGACCTGTTCGTTGCAACAAATTGATAAGCAATGCTTTCTTATAATGCCAACTTTGTACAAGAAAGCTGGGTCCCCAATACTTGTATGGCATACCAAATTTGCAGTTTGAAAGAAGTGTGCACAGTTGAATTGACATATTTATTTATTTTTCTAGAAAGCTAGAGTAACAGGATAAGTAGAGAGAAACAAATATTGAAGGCATGCAATAGAAATCATACCAGAAAGCAAATCGGGCACTCTTCACGATCGTAGGTGGTTTCTTCATCTCCGGGATAGCAAGGTGCTAACTTTGATTCTAGTATTAGCTTCCTCAGCTTCTTGTGATCCACATCTTTGTGATTATACAACCCTTGCGCCTCGTATATTTCTCATCTACCACTTGTCTCCTCCTCCCAAGTTTATTACCCATCCAACAAAACTTAATTCTTCTCGTCTGCCGACTATGTTCCTACTCTTAAACAAAATAAACTACCACAACAACAACAAAAATAACAAGCCTTCCTCTACAAAAACCACTCTCTCAACCACATCACGAAATCATCTTCACTGAACATAAGATTGTACTCAACAAACAACAATCTAACAGCCAAACCCCCCATCTACAACACAATCAACAGAAGTGANNANCCNNGNTNNNNNNNNNNNNNNNNNNNNNNNNNNNNNNNNNNNNNNNNNNNNNNNNNNNNNNNNNNNNNNNNNNNNNNNNNNNNNNNNNNNNNNNNNNNNNNNNNNNNN

>S6_5_19_2R_PREMIX Sample_Name=S6_5_2R_PREMIX Chromat_id=7231338 Read_id=7158981 Version=1 Length=729

NNNNNNNNNTNCGCTGGNTGGCAATAATGATTTNATTTTGACTGATAGTGACCTGTTCGTTGCAACAAATTGATAAGCAATGCTTTCTTATAATGCCAACTTTGTACAAGAAAGCTGGGTCCCCAATACTTGTATGGCATACCAAATTTGCAGTTTGAAATAAGTGTGAACAGTTGAATTGACATATTTGCTTCTTTTTTCTTTTTCTTTTTTTTTTTCTAAAAAGCTAGAGTAACAGGATAAGTAGAGAGAAACAAATATTAAAGGCATGCGATAGAAACCATACCAGAAAGCAAATCGGGCACTCTTCACGATCGTATGCGGTTTCTTCATCTCCGGGATAGCAAGGTGCTAACTTCGATTCTAGTATTAGCTTCCTCAGCTTCTTGTGATCCACATCTTTATGATTATACAACCCTTGCGGGCCTCGTATATTTCTCATCTACCACTTGTCTCCTCCTCCCAAGTTTATTACCCATCCAAAAATTCCAATTTTTCTCGTCCGCCGACTATGTTCCGAACTCTTCAACAAAATAAACCAACACAACAACAACAACAACAACAAAAGTAACAAACCTTCCACTACAAAAACCACTCACTCAACCACATCACAAAATCATCTTCACTCAAAACCAAATTACGTTAACGCCACACTCAACAAACCAACACAATCAACAGANNNGGNNNNCCNTTTTTTANNNNNNNNNNNNNNNNNNAAAAAAAANNAAA

>S6_6_2R_PREMIX Sample_Name=S6_6_2R_PREMIX Chromat_id=7158992 Read_id=7096207 Version=1 Length=673

GGCGAGCCTGCCGCTGGATGGCAATAATGATTTTATTTTGACTGATAGTGACCTGTTCGTTGCAACAAATTGATAAGCAATGCTTTCTTATAATGCCAACTTTGTACAAGAAAGCTGGGTCCCCAATACTTGTATGGCATACCAAATTTGCAGTTTGAAAGAAGTGTGCACAGTTGAATTGACATATTTATTTATTTTTCTAGAAAGCTAGAGTAACAGGATAAGTAGAGAGAAACAAATATTGAAGGCATGCAATAGAAATCATACCAGAAAGCAAATCGGGCACTCTTCACGATCGTAGGTGGTTTCTTCATCTCCGGGATAGCAAGGTGCTAACTTTGATTCTAGTATTAGCTTCCTCAGCTTCTTGTGATCCACATCTTTGTGATTATACAACCCTTGCGCCTCGTATATTTCTCATCTACCACTTGTCTCCTCCTCCCAAGTTTATTACCCATCCAACAAAACTTAATTCTTCTCGTCTGCCGACTATGTTCCTACTCTTAAACAAAATAAACTACCACAACAACAACAAAAATAACAAGCCTTCCTCTACAAAAACCACTCTCTCAACCACATCACGAAATCATCTTCACTGAACATAAGATTGTACTCAACAAACAACAATCTAACAGCCAAACCCCCCATCTACAACACAATCAACAGAAGAAAA

>S6_6_19_2R_PREMIX Sample_Name=S6_6_2R_PREMIX Chromat_id=7231339 Read_id=7158987 Version=1 Length=698

NNNNNNNNNTNNNCTGGNTGGNAATAATGATTTNATTTTGACTGATAGTGACCTGTTCGTTGCAACAAATTGATAAGCAATGCTTTCTTATAATGCCAACTTTGTACAAGAAAGCTGGGTCCCCAATACTTGTATGGCATACCAAATTTGCAGTTTGAAATAAGTGTGAACAGTTGAATTGACATATTTATTTATTTTTCTAGAAAGCTAGAGTAACAGGATAAGTAGAGAGAAACAAATATTGAAGGCATGCAATAGAAATCATACCAGAAAGCAAATCGGGCACTCTTCACGATCGTAGGTGGTTTCTTCATCTCCGGGATAGCAAGGTGCTAACTTTGATTCTAGTATTAGCTTCCTCAGCTTCTTGTGATCCACATCTTTGTGATTATACAACCCTTGCGCCTCGTATATTTCTCATCTACCACTTGTCTCCTCCTCCCAAGTTTATTACCCATCCAACAAAACTTAATTCTTCTCGTCTGCCGACTATGTTCCTACTCTTAAACAAAATAAACTACCACAACAACAACAAAAATAACAAGCCTTCCTCTACAAAAACCACTCTCTCAACCACATCACGAAATCATCTTCACTGAACATAAGATTGTACTCAACAAACAACAATCTAACAGCCAAACCCCCCATCTACAACACAATCAACAGAAGGNAAAAANCCTTGTTANNNNNNNNNNNNA

>S6_7_2R_PREMIX Sample_Name=S6_7_2R_PREMIX Chromat_id=7158993 Read_id=7096187 Version=1 Length=1065

GTCAGCCTGCGCTGGATGGCAATAATGATTTTATTTTGACTGATAGTGACCTGTTCGTTGCAACAAATTGATAAGCAATGCTTTCTTATAATGCCAACTTTGTACAAGAAAGCTGGGTCCCCAATACTTGTATGGCATACCAAATTTGCAGTTTGAAAGAAGTGTGCACAGTTGAATTGACATATTTATTTATTTTTCTAGAAAGCTAGAGTAACAGGATAAGTAGAGAGAAACAAATATTGAAGGCATGCAATAGAAATCATACCAGAAAGCAAATCGGGCACTCTTCACGATCGTAGGTGGTTTCTTCATCTCCGGGATAGCAAGGTGCTAACTTTGATTCTAGTATTAGCTTCCTCAGCTTCTTGTGATCCACATCTTTGTGATTATACAACCCTTGCGGGCCTCGTATATTTCTCATCTACCACTTGTCTCCTCCTCCCAAGTTTATTACCCATCCAACAAAACTTAATTCTTCTCGTCTGCCGACTATGTTCCTACTCTTAAACAAAATAAACTACCACAACAACAACAAAAATAACAAGCCTTCCTCTACAAAAACCACTCTCTCAACCACATCACGAAATCATCTTCACTGAACATAAGATTGTACTCAACAAACAACAATCTAACAGCCAAACCCCCCATCTACAACACAATCAACAGAGTGAAAAAACACCTGTTATAGTAATTGTCTTTGGGGGGTTTTGTCTTTCAATTCTGGTTTTTTTTGAAAAAGAACTTCTCGGCTGAGGTATGTTTTTTTTTTTGGTTTGGAACTGGATTTAGATTTGGATGTGATTTTTTTTATTTTTCCTGTTCCATTTTTCTTTTGTATATTTTGATTTTACAATTAGGAAGAGAAAATGAAGTTTTTTTATGTTCGATTTAATTCCTCACGTTTTCCTTTGTTCTTATTTGAAATCTGTCCCCCCTACCTGGGTTTGAGGCCCTGAATGTCCACTTGTATGTTACTAATATTGCTCAACATTAGTACCCTCTATGCTTCATCCCTCCAGTAAGTTGACATAACAGAATATCAAATACATGAAGAGTCATCTAGAG

>S6_7_19_2R_PREMIX Sample_Name=S6_7_2R_PREMIX Chromat_id=7231340 Read_id=7158984 Version=1 Length=718

NNNNNNNNNCTNCNCTGGNNGGCAATAATGATTTNATTTTGACTGATAGTGACCTGTTCGTTGCAACAAATTGATAAGCAATGCTTTCTTATAATGCCAACTTTGTACAAGAAAGCTGGGTCCCCAATACTTGTATGGCATACCAAATTTGCAGTTTGAAAGAAGTGTGCACAGTTGAATTGACATATTTATTTATTTTTCTAGAAAGCTAGAGTAACAGGATAAGTAGAGAGAAACAAATATTGAAGGCATGCAATAGAAATCATACCAGAAAGCAAATCGGGCACTCTTCACGATCGTAGGTGGTTTCTTCATCTCCGGGATAGCAAGGTGCTAACTTTGATTCTAGTATTAGCTTCCTCAGCTTCTTGTGATCCACATCTTTGTGATTATACAACCCTTGCGGCCTCGTATATTTCTCATCTACCACTTGTCTCCTCCTCCCAAGTTTATTACCCATCCAAAAATTCCAATTTTTCTCGTCCGCCGACTATGTTCCGAACTCTTCAACAAAATAAACCAACACAACAACAACAACAACAACAAAAGTAACAAACCTTCCACTACAAAAACCACTCACTCAACCACATCACAAAATCATCTTCACTCAAAACCAAATTACGTTAACGCCACACTCAACAAACCAACACAATCAACAGAAGNNNNNNNNCCTTTTTANNNNNNNNNNAAAANNNNNNAAANNNNNNNNAAANNNNNA

>S6_8_2R_PREMIX Sample_Name=S6_8_2R_PREMIX Chromat_id=7158998 Read_id=7096214 Version=1 Length=669

GAAAAGCTGCGCTGGATGGCAATAATGATTTTATTTTGACTGATAGTGACCTGTTCGTTGCAACAAATTGATAAGCAATGCTTTCTTATAATGCCAACTTTGTACAAGAAAGCTGGGTCCCCAATACTTGTATGGCATACCAAATTTGCAGTTTGAAAGAAGTGTGCACAGTTGAATTGACATATTTATTTATTTTTCTAGAAAGCTAGAGTAACAGGATAAGTAGAGAGAAACAAATATTGAAGGCATGCAATAGAAATCATACCAGAAAGCAAATCGGGCACTCTTCACGATCGTAGGTGGTTTCTTCATCTCCGGGATAGCAAGGTGCTAACTTTGATTCTAGTATTAGCTTCCTCAGCTTCTTGTGATCCACATCTTTGTGATTATACAACCCTTGCGCCTCGTATATTTCTCATCTACCACTTGTCTCCTCCTCCCAAGTTTATTACCCATCCAACAAAACTTAATTCTTCTCGTCTGCCGACTATGTTCCTACTCTTAAACAAAATAAACTACCACAACAACAACAAAAATAACAAGCCTTCCTCTACAAAAACCACTCTCTCAACCACATCACGAAATCATCTTCACTGAACATAAGATTGTACTCAACAAACAACAATCTAACAGCCAAACCCCCCATCTACAACACAATCAACAGAAGTG

>S6_8_19_2R_PREMIX Sample_Name=S6_8_2R_PREMIX Chromat_id=7231341 Read_id=7158977 Version=1 Length=702

NNNNNNNNCTNCNCTGGNNGGCAANANTGATTTNATTTTGACTGATAGTGACCTGTTCGTTGCAACAAATTGATAAGCAATGCTTTCTTATAATGCCAACTTTGTACAAGAAAGCTGGGTCCCCAATACTTGTATGGCATACCAAATTTGCAGTTTGAAATAAGTGTGCACAGTTGAATTGACATATTNNNTTATTTTTNNNNAANNNTNNNTNNNCNNNANAAGTAGAGAGAAACGNNTATNNANGGCNACAANNTANNNNATNCCAGAAANNAANNNNNNCACTCTNNACGATCGTACGCNNNNNNTTCNNNTCCGGNANNNNNNNNCGCTNCNTGCAATNNNNGNNNNNNNTTCCTCNNCTTCNNNTNNCCCNNACCTCTACGAGTATACCACCCTTGCNTNNNANNNNNNNNNNCGACCACTTGTCTCCTCCTCNNNCGACTATTACCCACCCCNAANTTCCTTTTTTACCNNTCCGCNNNCTATGNNNNNANCTCTCNACCAAAATAGACCCACACTNNNNCAACAACTACACCAAAACAACNACACCTTCCACTACAAAAACCACTCCCTCNCNNACATCAAACANNNATCTTCACCNNATACCAAATTACTCTTNCCCCACAATCAACTTACCATCACNCNCAACNCAANNNNAANNCCTGTNNANNAAGNTGAAAACCTGTTANNANNNNNCNT

>S6_11_2R_PREMIX Sample_Name=S6_11_2R_PREMIX Chromat_id=7231326 Read_id=7158992 Version=1 Length=676

NNNNNNNNNNTGCGCTGGNNGGCAATAATGATTTNATTTTGACTGATAGTGACCTGTTCGTTGCAACAAATTGATAAGCAATGCTTTCTTATAATGCCAACTTTGTACAAGAAAGCTGGGTCCCCAATACTTGTATGGCATACCAAATTTGCAGTTTGAAAGAAGTGTGCACAGTTGAATTGACATATTTATTTATTTTTCTAGAAAGCTAGAGTAACAGGATAAGTAGAGAGAAACAAATATTGAAGGCATGCAATAGAAATCATACCAGAAAGCAAATCGGGCACTCTTCACGATCGTAGGTGGTTTCTTCATCTCCGGGATAGCAAGGTGCTAACTTTGATTCTAGTATTAGCTTCCTCAGCTTCTTGTGATCCACATCTTTGTGATTATACAACCCTTGCCTCGTATATTTCTCATCTACCACTTGTCTCCTCCTCCCAAGTTTATTACCCATCCAAAAATTCCAATTTTTCTCGTCCGCCGACTATGTTCCGAACTCTTCAACAAAATAAACCAACACAACAACAACAACAACAACAAAAGTAACAAACCTTCCACTACAAAAACCACTCACTCAACCACATCACAAAATCATCTTCACTCAAAACCAAATTACGTTAACGCCACACTCAACAAACCAACACAATCAACAGAAGTNNANNNCCNNNTTNNA

>S6_12_2R_PREMIX Sample_Name=S6_12_2R_PREMIX Chromat_id=7231327 Read_id=7158982 Version=1 Length=690

NNNNNNNNNCNNNNCTGGATGGNAATAATGATTTNATTTTGACTGATAGTGACCTGTTCGTTGCAACAAATTGATAAGCAATGCTTTCTTATAATGCCAACTTTGTACAAGAAAGCTGGGTCCCCAATACTTGTATGGCATACCAAATTTGCAGTTTGAAATAAGTGTGAACAGTTGAATTGACATATTTGCTTCTTTTTTCTTTTTCTTTTTTTTTTCTAAAAAGCTAGAGTAACAGGATAAGTAGAGAGAAACAAATATTAAAGGCATGCGATAGAAACCATACCAGAAAGCAAATCGGGCACTCTTCACGATCGTATGCGGTTTCTTCATCTCCGGGATAGCAAGGTGCTAACTTCGATTCTAGTATTAGCTTCCTCAGCTTCTTGTGATCCACATCTTTATGATTATACAACCCTTGCTATATTTCTCATCTACCACTTGTCTCCTCCTCCCAAGTTTATTACCCATCCAAAAATTCCAATTTTTCTCGTCCGCCGACTATGTTCCGAACTCTTCAACAAAATAAACCAACACAACAACAACAACAACAACAAAAGTAACAAACCTTCCACTACAAAAACCACTCACTCAACCACATCACACAATCATCTTCACTCAAAACCAAATTACGTTAACGCCACACTCAACAAACCAACACAATCAACAGAANNGGGAANNCCNGTTAAA

>S6_13_2R_PREMIX Sample_Name=S6_13_2R_PREMIX Chromat_id=7231328 Read_id=7158993 Version=1 Length=697

NNNNNNNNNTGNNNCTGGNNGGCAATAATGATTTNATTTTGACTGATAGTGACCTGTTCGTTGCAACAAATTGATAAGCAATGCTTTCTTATAATGCCAACTTTGTACAAGAAAGCTGGGTCCCCAATACTTGTATGGCATACCAAATTTGCAGTTTGAAATAAGTGTGAACAGTTGAATTGACATATTTGCTTCTTTTTTCTTTTTCTTTTTTTTTTCTAAAAAGCTAGAGTAACAGGATAAGTAGAGAGAAACAAATATTAAAGGCATGCGATAGAAACCATACCAGAAAGCAAATCGGGCACTCTTCACGATCGTATGCGGTTTCTTCATCTCCGGGATAGCAAGGTGCTAACTTCGATTCTAGTATTAGCTTCCTCAGCTTCTTGTGATCCACATCTTTATGATTATACAACCCTTGCGGGCCTCGTATATTTCTCATCTACCACTTGGCTCCTCCTCCCAAGTTTATTACCCATCCAAAAATTCCAATTTTTCTCGTCCGCCGACTATGTTCCGAACTCTTCAACAAAATAAACCAACACAACAACAACAACAACAACAAAAGTAACAAACCTTCCACTACAAAAACCACTCACTCAACCACATCACAAAATCATCTTCACTCAAAACCAAATTACGTTAACGCCACACTCAACAAACCAACACAATCAACAGAANNGGGANNCCCNTTTTT

>S6_14_2R_PREMIX Sample_Name=S6_14_2R_PREMIX Chromat_id=7231329 Read_id=7158991 Version=1 Length=692

NNNNNNNNNCTGCGCTGGATGGNAATAATGNTTTNATTTTGACTGATAGTGACCTGTTCGTTGCAACAAATTGATAAGCAATGCTTTCTTATAATGCCAACTTTGTACAAGAAAGCTGGGTCCCCAATACTTGTATGGCATACCAAATTTGCAGTTTGAAATAAGTGTGAACAGTTGAATTGACATATTTGCTTCTTTTTTCTTTTTCTTTTTTTTTTCTAAAAAGCTAGAGTAACAGGATAAGTAGAGAGAAACAAATATTAAAGGCATGCGATAGAAACCATACCAGAAAGCAAATCGGGCACTCTTCACGATCGTATGCGGTTTCTTCATCTCCGGGATAGCAAGGTGCTAACTTCGATTCTAGTATTAGCTTCCTCAGCTTCTTGTGATCCACATCTTTATGATTATACAACCCTTGCTATATTTCTCATCTACCACTTGTCTCCTCCTCCCAAGTTTATTACCCATCCAAAAATTCCAATTTTTCTCGTCCGCCGACTATGTTCCGAACTCTTCAACAAAATAAACCAACACAACAACAACAACAACAACAAAAGTAACAAACCTTCCACTACAAAAACCACTCACTCAACCACATCACAAAATCATCTTCACTCAAAACCAAATTACGTTAACGCCACACTCAACAAACCAACACAATCAACAGAAGTGAAANNCNNTNGTTANNA

>S6_15_2R_PREMIX Sample_Name=S6_15_2R_PREMIX Chromat_id=7231330 Read_id=7158980 Version=1 Length=694

NNNNNNNNNNTNNNCTGGATGGCAATAATGATTTTATTTTGACTGATAGTGACCTGTTCGTTGCAACAAATTGATAAGCAATGCTTTCTTATAATGCCAACTTTGTACAAGAAAGCTGGGTCCCCAATACTTGTATGGCATACCAAATTTGCAGTTTGAAATAAGTGTGAACAGTTGAATTGACATATTTGCTTCTTTTTTCTTTTTCTTTTTTTTTTCTAAAAAGCTAGAGTAACAGGATAAGTAGAGAGAAACAAATATTAAAGGCATGCGATAGAAACCATACCAGAAAGCAAATCGGGCACTCTTCACGATCGTATGCGGTTTCTTCATCTCCGGGATAGCAAGGTGCTAACTTCGATTCTAGTATTAGCTTCCTCAGCTTCTTGTGATCCACATCTTTATGATTATACAACCCTTGCGGGCCTCGTATATTTCTCATCTACCACTTGTCTCCTCCTCCCAAGTTTATTACCCATCCAAAAATTCCAATTTTTCTCGTCCGCCGACTATGTTCCGAACTCTTCAACAAAATAAACCAACACAACAACAACAACAACAACAAAAGTAACAAACCTTCCACTACAAAAACCACTCACTCAACCACATCACAAAATCATCTTCACTCAAAACCAAATTACGTTAACGCCACACTCAACAAACCAACACAATCAACAGAGTGNNNAANCNNNTT

>S6_16_2R_PREMIX Sample_Name=S6_16_2R_PREMIX Chromat_id=7231331 Read_id=7158990 Version=1 Length=685

NNNNNNNNNNNNNCTGGATGGCAATAATGATTTTATTTTGACTGATAGTGACCTGTTCGTTGCAACAAATTGATAAGCAATGCTTTCTTATAATGCCAACTTTGTACAAGAAAGCTGGGTCCCCAATACTTGTATGGCATACCAAATTTGCAGTTTGAAAGAAGTGTGCACAGTTGAATTGACATATTTATTTATTTTTCTAGAAAGCTAGAGTAACAGGATAAGTAGAGAGAAACAAATATTGAAGGCATGCAATAGAAATCATACCAGAAAGCAAATCGGGCACTCTTCACGATCGTAGGTGGTTTCTTCATCTCCGGGATAGCAAGGTGCTAACTTTGATTCTAGTATTAGCTTCCTCAGCTTCTTGTGATCCACATCTTTGTGATTATACAACCCTTGCGCCTCGTATATTTCTCATCTACCACTTGTCTCCTCCTCCCAAGTTTATTACCCATCCAACAAAACTTAATTCTTCTCGTCTGCCGACTATGTTCCTACTCTTAAACAAAATAAACTACCACAACAACAACAAAAATAACAAGCCTTCCTCTACAAAAACCACTCTCTCAACCACATCACGAAATCATCTTCACTGAACATAAGATTGTACTCAACAAACAACAATCTAACAGCCAAACCCCCCATCTACAACACAATCAACAGANGNNAAAANCNNNTGTTA

>S6_17_2R_PREMIX Sample_Name=S6_17_2R_PREMIX Chromat_id=7231332 Read_id=7158986 Version=1 Length=728

NNNNNNNNNNTNCGCTGGNTGGNAATAATGATTTNATTTTGACTGATAGTGACCTGTTCGTTGCAACAAATTGATAAGCAATGCTTTCTTATAATGCCAACTTTGTACAAGAAAGCTGGGTCCCCAATACTTGTATGGCATACCAAATTTGCAGTTTGAAATAAGTGTGAACAGTTGAATTGACATATTTGCTTCTTTTTTCTTTTTCTTTTTTTTTTCTAAAAAGCTAGAGTAACAGGATAAGTAGAGAGAAACAAATATTAAAGGCATGCGATAGAAACCATACCAGAAAGCAAATCGGGCACTCTTCACGATCGTATGCGGTTTCTTCATCTCCGGGATAGCAAGGTGCTAACTTCGATTCTAGTATTAGCTTCCTCAGCTTCTTGTGATCCACATCTTTATGATTATACAACCCTTGCGGGCCTCGTATATTTCTCATCTACCACTTGTCTCCTCCTCCCAAGTTTATTACCCATCCAAAAATTCCAATTTTTCTCGTCCGCCGACTATGTTCCGAACTCTTCAACAAAATAAACCAACACAACAACAACAACAACAACAAAAGTAACAAACCTTCCACTACAAAAACCACTCACTCAACCACATCACAAAATCATCTTCACTCAAAACCAAATTACGTTAACGCCACACTCAACAAACCAACACAATCAACAGAAGNNAAAANNNTNGGTNANNANNNNNNNNNNNNNNNNNAAAAAANNAAA

>S6_18_2R_PREMIX Sample_Name=S6_18_2R_PREMIX Chromat_id=7231333 Read_id=7158989 Version=1 Length=728

NNNNNNNNNTNNNCTGGATGGCAATAATGATTTNATTTTGACTGATAGTGACCTGTTCGTTGCAACAAATTGATAAGCAATGCTTTCTTATAATGCCAACTTTGTACAAGAAAGCTGGGTCCCCAATACTTGTATGGCATACCAAATTTGCAGTTTGAAATAAGTGTGAACAGTTGAATTGACATATTTGCTTCTTTTTTCTTTTTCTTTTTTTTTTCTAAAAAGCTAGAGTAACAGGATAAGTAGAGAGAAACAAATATTAAAGGCATGCGATAGAAACCATACCAGAAAGCAAATCGGGCACTCTTCACGATCGTATGCGGTTTCTTCATCTCCGGGATAGCAAGGTGCTAACTTCGATTCTAGTATTAGCTTCCTCAGCTTCTTGTGATCCACATCTTTATGATTATACAACCCTTGCGCCTCGTATATTTCTCATCTACCACTTGTCTCCTCCTCCCAAGTTTATTACCCATCCAAAAATTCCAATTTTTCTCGTCCGCCGACTATGTTCCGAACTCTTCAACAAAATAAACCAACACAACAACAACAACAACAACAAAAGTAACAAACCTTCCACTACAAAAACCACTCACTCAACCACATCACAAAATCATCTTCACTCAAAACCAAATTACGTTAACGCCACACTCAACAAACCAACACAATCAACAGAAGTNNNAAAACCCTTGNNNANNANNNNNNNNNNNNNNNNNAANNNNNNAAAA

PDS target

>322_T7_PREMIX Sample_Name=322_T7_PREMIX Chromat_id=6707478 Read_id=6646688 Version=1 Length=1264

NNNNNNNNNNTNNNNGCNNNNGCCGCCATGGCGGCCGCGGGAATTCGATTGCTTTGCTTGAGAAAAGCTCTCTTTTTCCCGTTTAGGATCTTGTTTATTTGCTTTCGTTTTTCTACTCGTTTGAATTTTAACTTGATTTTGTGGGTGAAGGCTAATTTTTCTCATAGTGTAAGAACAAGTTTCATATGTACTGTAAAAGCTAGAATCTTTTTTACTTTTGCATATAAATTTGTGTAATAAATGCTTAAGAACCAGAATATTTGAAACAGATAAGGAATTTTGCATAGTATTTAGGTTCACAAGTGGGACAATCTTCTTACACTGAAATATCTTTATGTCAGGCTTAATTTACTGCTATCTTGTTCAATAAAATGCCCCAAATTGGACTTGTTTCTGCCGTTAATTTGAGACCAAGGTAATTCAGCTTATCTTTGGAGCTCGAGGTCTTCGTTGGGAACTGAAAGTCAAGATGTTTGCTTGCAAAGGAATTTGTTATGTAATCACTAGTGAATTCGCGGCCGCCTGCAGGTCGACCATATGGGAGAGCTCCCAACGCGTTGGATGCATAGCTTGAGTATTCTATAGTGTCACCTAAATAGCTTGGCGTAATCATGGTCATAGCTGTTTCCTGTGTGAAATTGTTATCCGCTCACAATTCCACACAACATACGAGCCGGAAGCATAAAGTGTAAAGCCTGGGGTGCCTAATGAGTGAGCTAACTCACATTAATTGCGTTGCGCTCACTGCCCGCTTTCCAGTCGGGAAACCTGTCGTGCCAGCTGCATTAATGAATCGGCCAACGCGCGGGGAGAGGCGGTTTGCGTATTGGGCGCTCTTCCGCTTCCTCGCTCACTGACTCGCTGCGCTCGGTCGTTCGGCTGCGGCGAGCGGTATCAGCTCACTCAAAGGCGGTAATACGGTTATCCACAGAATCAGGGGATAACGCAGGAANGAACATGTGAGCAAAAGGCCAGCAAAAGGCCAGGAACCGTAAAAAGGCNGNNTTGCTGGGNTTTTTCCTAAGGTCCGCCCCCNTGAAGAGAANNANAAAANTCGACCNTCAATNNANGNNTGGNNAAACCCCCNGGGNTNNAAAANNNNNNNGGNTTNCCCCNGGAGNNCNTNNTGGGGNCTNNTTTNNNNCNNNGCNNNNANNNNGANNNNNNNNNTTNNNTNCTTGNNGNNGGGGGGNTCTCNNNNNNNCACNNTNNNNNNAATCCNNGNNNNNNNNNNNNCCTNNGANNNGTGGNNNNNNNNNNNNNN

>323_T7_PREMIX Sample_Name=323_T7_PREMIX Chromat_id=6707484 Read_id=6646664 Version=1 Length=1231

NNNNNNNNNNNNNNGCTCCGGCCGCCATGGCGGCCGCGGGAATTCGATTACATAACAAATTCCTTTGCAAGCGACCATCTTGACTTTCAGTTCCCAAAGAAGACCTCGAGCTCCAAAGATAAGCTGAATTACCTTGGCAAATTAACGGCAGAAACAAGTCCAATTTGGGGCATTTTACTGAACAAAATAGCAGTAAATTAAGCCTGACATAAAGATATTTCACTGTAAGAAGATTGTCCCACTTGTGAACCTAAATACTATGTAGAATTCCTTTGTTTTTTCAATATTCTGGTTCTTAAGCATTTATTACACAAATTTATATGCAAAAGTAAAAAAGATTCTAGCTTTTACAGTCCATACGAAACTTGTTCTCGCACTATGAGTAAAGTTATAATTCACCCACAAAATCAAGTTAAAATTTTAACGAGTAGAAAAACGAAAGCAAATAAACAAGATCCTAAACGGGAAAAGGAGAGAGCTTTTCTCAAGCAAAGCAATCACTAGTGAATTCGCGGCCGCCTGCAGGTCGACCATATGGGAGAGCTCCCAACGCGTTGGATGCATAGCTAGAGTATTCTATAGTGTCACCTAAATAGCTTGGCGTAGTCATGGTCATAGCTGTTTCCTGTGTGAGATTGTTATCCGCTCACAATTCCACACAACATACGAGCCGGAAGCATAAAGTGTAAAGCCTGGGGTGCCTAAGGAGTGAGCTAACTCACATTAATTGCGTTGCGCTCACTGCCCGCTTTCCAGTCGGGAAACCTGTCGTGCCAGCTGCATTAATGAATCGGCCAACGCGCGGGGAGAGGCAGTTTGCGTATTGGGCGCTCTTCCGCTTCCTCGCTCACTGACTCGCTGCGCTCGGTCGTTCGGCTGCGGCNAGCGGTATCAGCTCACTCAAAGGCGGTAATACGGTTATCCACAGAATCAGGGGATAACGNAGGAAAGANCATGTGAGCAAAAGGCCAGCGAANGGCCAGGAACCGTAAAAAGGCNGCGTTGCTGGCNTTTTNCNTNAGGCTCCNNCCCNTGACCAGGATCANAAAANTNNAACCTCAAGTCAANGNNGGNAAACCNANNNNNNCATAAANNTNNANNGNNTTNCNNNTGGANNNCNNNNNCCGGTCTTCNNGTNNNNNCNGNCCTCAANGGAAAAANGGGNCNNNNNNCCNTTTNGGAAANGGNNNGNTTNCNNNAANNNNNAGGGNNTTTTNNNNNNNNNNNNNNN

>327_T7_PREMIX Sample_Name=327_T7_PREMIX Chromat_id=6707483 Read_id=6646660 Version=1 Length=1198

NNNNNNNNNNNCNGCTCCGGCCGCCATGGCGGCCGCGGGAATTCGATTGCTTTGCTTGAGAAAAGCTCTCTTTTTCCCGTTTAGGATCTTGTTTATTTGCTTTCGTTTTTCTACTCGTTTGAATTTTAACTTGATTTTGTGGGTGAAGGCTAATTTTTCTCATAGTGTAAGAACAAGTTTCATATGTACTGTAAAAGCTAGAATCTTTTTTACTTTTGCATATAAATTTGTGTAATAAATGCTTAAGAACCAGAATATTTGAAAAAGATAAGGAATTTTGCATAGTATTTAGGTTCACAAGTGGGACAATCTTCTTACACTGAAATATCTTTATGTCAGGCTTAATTTACTGCTATCTTGTTCAATAAAATGCCCCAAATTGGACTTGTTTCTGCCGTTAATTTCCAAGGTAATTCAGCTTATCTTTGGAGCTCGAGGTCTTCTTTGGGAACTGAAAGTCAAGATGGTCGCTTGCAAAGGAATTTGTTATGTAATCACTAGTGAATTCGCGGCCGCCTGCAGGTCGACCATATGGGAGAGCTCCCAACGCGTTGGATGCATAGCTTGAGTATTCTATAGTGTCACCTAAATAGCTTGGCGTAATCATGGTCATAGCTGTTTCCTGTGTGAAATTGTTATCCGCTCACAATTCCACACAACATACGAGCCGGAAGCATAAAGTGTAAAGCCTGGGGTGCCTAATGAGTGAGCTAACTCACATTAATTGCGTTGCGCTCACTGCCCGCTTTCCAGTCGGGAAACCTGTCGTGCCAGCTGCATTAATGAATCGGCCAACGCGCGGGGAGAGGCGGTTTGCGTATTGGGCGCTCTTCCGCTTCCTCGCTCACTGACTCGCTGCGCTCGGTCGTTCGGCTGCGGCGAGCGGTATCAGCTCACTCAAAGGCGGTAATACGGTTATCCACAGAATCAGGGGATAACGCAGGAANGAACATGTGAGCAAAAGGCCAGCAAAAGGCCAGGAACCGTAAAAAGGCNNNTTNGCTGGGNTTTTTCCAANGGNTCNNCCCCCNTGAGAAGNNNNACAAAANTCGACCTCCAATTCAANGTGGNNAAACCCCNNNNNNTTNAAAAAANCNNGGNTTNCCCCNGGAGAACCCCNTGGGGNNNNNNGTTCCAACNNNNNGNAANNNAANNNNNNNCNNTCNCNNNNNNNNNNNGGNCNNTTTNNNNNNNNNNN

>434_T7_PREMIX Sample_Name=434_T7_PREMIX Chromat_id=6707491 Read_id=6646658 Version=1 Length=1326

NNNNNCNNNNNNNGCNNNNGCCGCCATGGCGGCCGCGGGAATTCGATTACATAACAAATTCCTTTGCAAGCGACCATCTTGACTTTCAGTTCCCAAAGAAGACCTCGAGCTCCAAAGATAAGCTGAATTACCTTGGAACTCTCAAATTAACGGCAGAAACAAGTCCAATTTGGGGCATTTTACTGAACAAAATAGCAGTAAATTAAGCCTGACATAAAGATATTTCACTGTAAGAAGATTGTCCCACTTGTGAACCTAAATACTATGTAGAATTCCTTTGTTTTTTCAATATTCTGGTTCTTAAGCATTTATTACACAAATTTATATGCAAAAGTAAAAAAGATTCTAGCTTTTACAGTCCATACGAAACTTGTTCTCGCACTATGAGTAAAGTTATAATTCACCCACAAAATCAAGTTAAAATTTTAACGAGTAGAAAAACGAAAGCAAATAAACAAGATCAATCACTAGTGAATTCGCGGCCGCCTGCAGGTCGACCATATGGGAGAGCTCCCAACGCGTTGGATGCATAGCTTGAGTATTCTATAGTGTCACCTAAATAGCTTGGCGTAATCATGGTCATAGCTGTTTCCTGTGTGAAATTGTTATCCGCTCACAATTCCACACAACATACGAGCCGGAAGCATAAAGTGTAAAGCCTGGGGTGCCTAATGAGTGAGCTAACTCACATTAATTGCGTTGCGCTCACTGCCCGCTTTCCAGTCGGGAAACCTGTCGTGCCAGCTGCATTAATGAATCGGCCAACGCGCGGGGAGAGGCGGTTTGCGTATTGGGCGCTCTTCCGCTTCCTCGCTCACTGACTCGCTGCGCTCGGTCGTTCGGCTGCGGCGAGCGGTATCAGCTCACTCAAAGGCGGTAATACGGTTATCCACAGAATCAGGGGATAACGCAGGAANGAACATGTGAGCAAAAGGCCAGCAAAAGGCCAGGAACCGTAAAAAGGCNNNNTTGCTGGCGTTTTNCNNAANNTCCGCCCCCNTGACAANNTCCNAAAAATCGACGCTCANNNNAANNNNGGNAAACCNNNNNNNNNNAANGNNNCAAGGNNTNCCCCNGGAGNNCCCNNNNGNNNTTNNNNTGTTAACANNCGNNTAANNANAGAANGNNGCCNTTTNNNNNNNNNNGAGNGGGNNNTTTNNNNNNNNNNNNNNNNNNNNNNNNCNNNNNNGGNNTCNNNNNNNCNNNNNNGNNNNNNANCCCCNNNCNNNNNNNNNNNNTTNNNNNNANNNCNNNNANNNNCNTNNNANCNNNNNNCNNNNNNNCCNNNNNNNNNNNNNNNTGTNNNCGTGNNNNNGGNNNTNNNNN

>436_T7_PREMIX Sample_Name=436_T7_PREMIX Chromat_id=6707497 Read_id=6646653 Version=1 Length=1302

NNNNNNNNNNNNNGCNNNNGCCGCCATGGCGGCCGCGGGAATTCGATTACATAACAAATTCCTTTGCAAGCGACCATCTTGACTTTCAGTTCCCAAAGAAGACCTCGAGCTCCAAAGATAAGCTGAATTACCTTGGTCTCAAATTAACGGCAGAAACAAGTCCAATTTGGGGCATTTTACTGAACAAAATAGCAGTAAATTAAGCCTGACATAAAGATATTTCACTGTAAGAAGATTGCCCCACTTGTGAACCTAAATACTATGTAGAATTCCTTTGTTTTTTCAATATTCTGGTTCTTAAGCATTTATTACACAAATTTATATGCAAAAGTAAAAGAGATTCTAGCTTTTACAGTCCATACGAAACTTGTTCTCGCACTATGAGTAAAGTTATAATTCACCCACAAAATCAAGTTAAAATTTTAACGAGTAGAAAAACGAAAGCAAATAAACAAGATCCTAAACGGGAAAAGGAGAGAGCTTTTCTCAAGCAAAGCAATCACTAGTGAATTCGCGGCCGCCTGCAGGTCGACCATATGGGAGAGCTCCCAACGCGTTGGATGCATAGCTTGAGTATTCTATAGTGTCACCTAAATAGCTTGGCGTAATCATGGTCATAGCTGTTTCCTGTGTGAAATTGTTATCCGCTCACAATTCCACACAACATACGAGCCGGAAGCATAAAGTGTAAAGCCTGGGGTGCCTAATGAGTGAGCTAACTCACATTAATTGCGTTGCGCTCACTGCCCGCTTTCCAGTCGGGAAACCTGTCGTGCCAGCTGCATTAATGAATCGGCCAACGCGCGGGGAGAGGCGGTTTGCGTATTGGGCGCTCTTCCGCTTCCTCGCTCACTGACTCGCTGCGCTCGGTCGTTCGGCTGCGGCGAGCGGTATCAGCTCACTCAAAGGCGGTAATACGGTTATCCACAGAATCAGGGGATAACGCAGGAANGAACATGTGAGCAAAAGGCCAGCAAAAGGCCAGGAACCGTAAAAAGGCGNNNNNGCTGGGNTTTTTCCAANGGNTCCGCCCCCNTGNNNNNNTCCNAAAANTNNNCGCTCANTTCANNNNNGGNNAAACCNNCNGGNCNNNANNNNNNNNCGGGNTTNCCNTCGGAGNNNCNTNNGGGNNNNNNNNNNNNNNNNNNGCNNNNNANNAANANNNCCCCNTCNNNNNNNNNNNNNNNNNNNNGNNNCNNNNNNNNNNNNNNNNNNNNNNNNNNNNNNNNNNNNNNNNNNCNNNGGGNTGGGGNNNNNNNNNNNNCCCNNNNNNNNNNNNNNNNNNNNNNNNNNNNNGNNNNN

>438_T7_PREMIX Sample_Name=438_T7_PREMIX Chromat_id=6707496 Read_id=6646685 Version=1 Length=1268

NNNNNNNNNNNNNNGCTCCGGCCGCCATGGCGGCCGCGGGAATTCGATTGCTTTGCTTGAGAAAAGCTCTCTTTTTCCCGTTTAGGATCTTGTTTATTTGCTTTCGTTTTTCTACTCGTTTGAATTTTAACTTGATTTTGTGGGTGAAGGCTAATTTTTCTCATAGTGTAAGAACAAGTTTCATATGTACTGTAAAAGCTAGAATCTTTTTTACTTTTGCATATAAATTTGTGTAATAAATGCTTAAGAACCAGAATATTTGAAAAAGATAAGGAATTTTGCATAGTATTTAGGTTCACAAGTGGGACAATCTTCTTACACTGAAATATCTTTATGTCAGGCTTAATTTACTGCTATCTTGTTCAATAAAATGCCCCAAATTGGACTTGTTTCTGCCGTTAATTTGAGAGTACAAGGTAATTCAGCTTATCTTTGGAGCTCGAGGTCTTCTTTGGGAACTGAAAGTCAAGATGGTCGCTTGCAAAGGAATTTGTTATGTAATCACTAGTGAATTCGCGGCCGCCTGCAGGTCGACCATATGGGAGAGCTCCCAACGCGTTGGATGCATAGCTTGAGTATTCTATAGTGTCACCTAAATAGCTTGGCGTAATCATGGTCATAGCTGTTTCCTGTGTGAAATTGTTATCCGCTCACAATTCCACACAACATACGAGCCGGAAGCATAAAGTGTAAAGCCTGGGGTGCCTAATGAGTGAGCTAACTCACATTAATTGCGTTGCGCTCACTGCCCGCTTTCCAGTCGGGAAACCTGTCGTGCCAGCTGCATTAATGAATCGGCCAACGCGCGGGGAGAGGCGGTTTGCGTATTGGGCGCTCTTCCGCTTCCTCGCTCACTGACTCGCTGCGCTCGGTCGTTCGGCTGCGGCGAGCGGTATCAGCTCACTCAAAGGCGGTAATACGGTTATCCACAGAATCAGGGGATAACGCAGGAANGAACATGTGAGCAAAAGGCCAGCAAAAGGCCAGGAACCGTAAAAAGGCNGCGTTGCTGGCGTTTTTCCANNGGCTCCCCCCNNTGACANNNNNNNAAANTNNANNNNNNNNNNNNNNNNNNAANCCNNNNNGNATATAAAANTACNNGGNTTNCCCCNGGAANCCCCCCNGGNCNNNCNNGTGNCNCACNGGNCGNAANCGAGAANNTNGCCNTTTNNTNTTNGNNGNNGGGGNNTNNCNNNNNNNANCNNNNGNNNNNANCNCNNNTNNNNNNNNNNNACTNNNNNNNTNNNNNANCCNNNNNNNNNNCNTNN

>717_T7_PREMIX Sample_Name=717_T7_PREMIX Chromat_id=6707494 Read_id=6646690 Version=1 Length=1281

NNNNNNNNNNNCNGCTCCGGCCGCCATGGCGGCCGCGGGAATTCGATTACATAACAAATTCCTTTGCAAGCGACCATCTTGACTTTCAGTTCCCAAAGAAGACCTCGAGCTCCAAAGATAAGCTGAATTACCTTGGCAAATTAACGGCAGAAACAAGTCCAATTTGGGGCATTTTACTGAACAAAATAGCAGTAAATTAAGCCTGACATAAAGATATTTCACTGTAAGAAGATTGTCCCACTTGTGAACCTAAATACTATGTAGAATTCCTTTGTTTTTTCAATATTCTGGTTCTTAAGCATTTATTACACAAATTTATATGCAAAAGTAAAAAAGATTCTAGCTTTTACAGTCCATACGAAACTTGTTCTCGCACTATGAGTAAAGTTATAATTCACCCACAAAATCAAGTTAAAATTTTAACGAGTAGAAAAACGAAAGCAAATAAACAAGATCCTAAACGGGAAAAGGAGAGAGCTTTTCTCAAGCAAAGCAATCACTAGTGAATTCGCGGCCGCCTGCAGGTCGACCATATGGGAGAGCTCCCAACGCGTTGGATGCATAGCTTGAGTATTCTATAGTGTCACCTAAATAGCTTGGCGTAATCATGGTCATAGCTGTTTCCTGTGTGAAATTGTTATCCGCTCACAATTCCACACAACATACGAGCCGGAAGCATAAAGTGTAAAGCCTGGGGTGCCTAATGAGTGAGCTAACTCACATTAATTGCGTTGCGCTCACTGCCCGCTTTCCAGTCGGGAAACCTGTCGTGCCAGCTGCATTAATGAATCGGCCAACGCGCGGGGAGAGGCGGTTTGCGTATTGGGCGCTCTTCCGCTTCCTCGCTCACTGACTCGCTGCGCTCGGTCGTTCGGCTGCGGCGAGCGGTATCAGCTCACTCAAAGGCGGTAATACGGTTATCCACAGAATCAGGGGATAACGCAGGAANGAACATGTGAGCAAAAGGCCAGCAAAAGNNNAGGAACCGTAAAAAGGCCNGTTGCTGGGNTTTTNCNNNNNNCTCCNCCCCCNTGNNNANANTCANAAAANTNNNCCNTCAATTNANGNNTGGGNAAACCCCNNGGNNNNTAANNNTNCAAGGGTTTCTCTNGNAGNCNTNNNTGGNNNNNTNNNNCNNNNNCGNNNTAACANANNNNNNNNNNNCTNNNTCCGGAGNNNNNNNNNCNNNNCNNANNNNNNGNNNNNNNNNNNNNGNNNTGNNCCCCCNNCNNGGGNNNNANCCNNNNNNNCGNNNNNNNNNTNANNNNNNNNTTAGNNNNN

>732_T7_PREMIX Sample_Name=732_T7_PREMIX Chromat_id=6707501 Read_id=6646678 Version=1 Length=1383

NNNNNNNNNNNNNNNNCNNNNGCCGCCCTGGCGGCCGCGGGAATTCGATTACATAACAAATTCCTTTGCAAGCAAACATCTTGACTTTCAGTTCCCAACGAAGACCTCGAGCTCCAAAGATAAGCTGAATTACCTTGGAACACTCAAATTAACGGCAGAAATAAGTCCAATTTGGGGCATTTTATTGAACAAGATAGCAGTAAATTAAGCCTGACATAAAGATATTTCAGTGTAAGAAGATTGTCCCACTTGTGAACCTAAATACTATGCAAAATTCCCTATCTTTTTCAAATATTCTGGTTCTTAAGCATTTATTACACAAATTTATATGCAAAAGTAAAAAAGATTCTAGCTTTTACAGTACATATGAAACTTGTTCTTACACTATGAGAAAAATTAGCCTTCACCCACAAAATCAAGTTAAAATTCAAACGAGTAGAAAAACGAAAGCAAATAAACAAGATCCTAAACGGGAAAAAGAGAGCTTTTCTCAAGCAAAGCAATCACTAGTGAATTCGCGGCCGCCTGCAGGTCGACCATATGGGAGAGCTCCCAACGCGTTGGATGCATAGCTTGAGTATTCTATAGTGTCACCTAAATAGCTTGGCGTAATCATGGTCATAGCTGTTTCCTGTGTGAAATTGTTATCCGCTCACAATTCCACACAACATACGAGCCGGAAGCATAAAGTGTAAAGCCTGGGGTGCCTAATGAGTGAGCTAACTCACATTAATTGCGTTGCGCTCACTGCCCGCTTTCCAGTCGGGAAACCTGTCGTGCCAGCTGCATTAATGAATCGGCCAACGCGCGGGGAGAGGCGGTTTGCGTATTGGGCGCTCTTCCGCTTCCTCGCTCACTGACTCGCTGCGCTCGGTCGTTCGGCTGCGGCGAGCGGTATCAGCTCACTCAAAGGCGGTAATACGGTTATCCACAGAATCAGGGGATAACGCAGGAANGAACATGTGAGCAAAAGGCCAGCAAAAGGCCAGGAACCGTAAAAAGGCCGNNTTGCTGNNNTTTTTCCTANGGNTCCGCCCCCNTGNCANNGATCNNAAAANTNNNACNNNAANNNAANNNNNCGAAACCNNCCGGNCTTAAAAAANNNNNGGGNTTNCCCCCGNNNGNCCCNNNGGNNNNNNNNNTGTNNNNANNNGGNTNNNNGAAANNNNNCCCNTCNNNNTNNNNNNNGNNNNNNNNNNNNNNNNCNNNNNNNNNNNNNNNNNNNNNNNNNNNNNNCNNNCCNNNNNNGNNTNNNANNNNNNNTNNNNNNNNNNCNNNNNNNNNNNNCNNNGNNNNNNGNGANNNNNNNANNNNNNNNNNGTNNNNNNNNNNNANNNNNNNNNNTNGGGNNTNNNNNNNNNNNNNNTNNNAANNNNGNNNNNN

>733_T7_PREMIX Sample_Name=733_T7_PREMIX Chromat_id=6707499 Read_id=6646693 Version=1 Length=1349

NNNNNNNNNNNNNNGCTNNNGCCGCCATGGCGGCCGCGGGAATTCGATTCTTTGCTTGAGAAAAGCTCTCTTTTTCCCGTTTAGGATCTTGTTTATTTGCTTTCGTTTTTCTACTCGTTTGAATTTTAACTTGATTTTGTGGGTGAAGGCTAATTTTTCTCATAGTGTAAGAACAAGTTTCATATGTACTGTAAAAGCTAGAATCTTTTTTACTTTTGCATATAAATTTGTGTAATAAATGCTTAAGAACCAGAATATTTGAAAAAGATAAGGAATTTTGCATAGTATTTAGGTTCACAAGTGGGACAATCTTCTTACGCTGAAATATCTTTATGTCAGGCTTAATTTACTGCTATCTTGTTCAATAAAATGCCCCAAATTGGACTTGTTTCTGCCGTTAATTTGAGATCCAAGGTAATTCAGCTTATCTTTGGAGCTCGAGGTCTTCGTTGGGAACTGAAAGTCAAGATGTTTGCTTGCAAAGGAATTTGTTATGTAATCACTAGTGAATTCGCGGCCGCCTGCAGGTCGACCATATGGGAGAGCTCCCAACGCGTTGGATGCATAGCTTGAGTATTCTATAGTGTCACCTAAATAGCTTGGCGTAATCATGGTCATAGCTGTTTCCTGTGTGAAATTGTTATCCGCTCACAATTCCACACAACATACGAGCCGGAAGCATAAAGTGTAAAGCCTGGGGTGCCTAATGAGTGAGCTAACTCACATTAATTGCGTTGCGCTCACTGCCCGCTTTCCAGTCGGGAAACCTGTCGTGCCAGCTGCATTAATGAATCGGCCAACGCGCGGGGAGAGGCGGTTTGCGTATTGGGCGCTCTTCCGCTTCCTCGCTCACTGACTCGCTGCGCTCGGTCGTTCGGCTGCGGCGAGCGGTATCAGCTCACTCAAAGGCGGTAATACGGTTATCCACAGAATCAGGGGATAACGCAGGAANGAACATGTGAGCAAAAGGCCAGCAAAAGGCCAGGAACCGTAAAAAGGCCGNGTTGCTGGCGTTTTTCNNNNGGNTCCGCCCCCNTGACAAGATNNNNAAANTCGACGCTCAAGTCAAANTGNNNAANCCNNANGGANNNANNNNNCNNGGNNTTNCCCNNGNANNNCCCNTCNNNNNNNNNNNNNCNAACNTGCGNNNNNGNAAANNNNCCCNTTNCNNNNNNNNNNNGGNNNTTCNNNNNNCNNNNNNNNNNNNNNNNNNNNGNNNNNNNNNNNNNNNNNNGGNNNNNNNNNNCCCNNNNNNNNNNNNNNNNNNNNNNNNNNNNNGTTNACANNNNNNNANNTNNNNNNNTCNNNNAAGAACCNNNNGTGNNGATNNNNNNNNNNNNNNNNNNNAN

>748_T7_PREMIX Sample_Name=748_T7_PREMIX Chromat_id=6707505 Read_id=6646667 Version=1 Length=1350

NNNNNNNNNNNNNGCTCCGGCCGCCATGGCGGCCGCGGGAATTCGATTACATAACAAATTCCTTTGCAAGCAAACATCTTGACTTTCAGTTCCCAACGAAGACCTCGAGCTCCAAAGATAAGCTGAATTACCTTGGACTCTCAAATTAACGGCAGAAACAAGTCCAATTTGGGGCATTTTATTGAACAAGATAGCAGTAAATTAAGCCTGACATAAAGATATTTCAGTGTAAGAAGATTGTCCCACTTGTGAACCTAAATACTATGCAAAATTCCTTATCTTTTTCAAATATTCTGGTTCTTAAGCATTTATTACACAAATTTATATGCAAAAGTAAAAAAGATTCTAGCTTTTACAGTACATATGAAACTTGTTCTTACACTATGAGAAAAATTAGCCTTCACCCACAAAATCAAGTTAAAATTCAAACGAGTAGAAAAACGAAAGCAAATAAACAAGATCCTAAACGGGAAAAAGAGAGCTTTTCACAAGCAAAGCAATCACTAGTGAATTCGCGGCCGCCTGCAGGTCGACCATATGGGAGAGCTCCCAACGCGTTGGATGCATAGCTTGAGTATTCTATAGTGTCACCTAAATAGCTTGGCGTAATCATGGTCATAGCTGTTTCCTGTGTGAAATTGTTATCCGCTCACAATTCCACACAACATACGAGCCGGAAGCATAAAGTGTAAAGCCTGGGGTGCCTAATGAGTGAGCTAACTCACATTAATTGCGTTGCGCTCACTGCCCGCTTTCCAGTCGGGAAACCTGTCGTGCCAGCTGCATTAATGAATCGGCCAACGCGCGGGGAGAGGCGGTTTGCGTATTGGGCGCTCTTCCGCTTCCTCGCTCACTGACTCGCTGCGCTCGGTCGTTCGGCTGCGGCGAGCGGTATCAGCTCACTCAAAGGCGGTAATACGGTTATCCACAGAATCAGGGGATAACGCAGGAANGAACATGTGAGCAAAAGGCCAGCAAAAGGCCAGGAACCGTAAAAAGGCNGNNTTGCTGGCGTTTTTCCAANGGNNCCGCCCCCNTGACAAGCNCCNAAAAATCGACGCTCAAGTCAANNNNNNNAANCCNGANGGNNTNNAANGATACNNGGGNTTNCCCNNNNNNNNNCNNNNGGGNTCTCCTNNNNNNNNNNNCNNTNNNNNAANNNNNNCCCNTTTNNNNNNNNNNNNGGGGNCGTTNCNNNNNNNCNNNNGGGNNNNNNCCNNNNNGGNNNNNNNGCCNNNNNNGGNNNNNNNANNCCNNNNNNNNNNNNNNNNNNNNNNNNNNNNNNNNNNTNNNNNNNNNNNNGANNANNCNNNNANTNNNNNNNAANGNNNNTGNNNNNAGNNNNNNNNN

>762_T7_PREMIX Sample_Name=762_T7_PREMIX Chromat_id=6707507 Read_id=6646740 Version=1 Length=1313

NNNNNCNNNNNNNGCTNNNGCCGCCATGGCGGCCGCGGGAATTCGATTGCTTTGCTTGAGAAAAGCTCTCTTTTTCCCGTTTAGGATCTTGTTTATTTGCTTTCGTTTTTCTACTCGTTTGAATTTTAACTTGATTTTGTGGGTGAAGGCTAATTTTTCTCATAGTGTAAGAACAAGTTTCATATGTACTGTAAAAGCTAGAATCTTTTTTACTTTTGCATATAAATTTGTGTAATAAATGCTTAAGAACCAGGATATTTGAAAAAGATAAGGAATTTTGCATAGTATTTAGGTTCACAAGTGGGACAATCTTCTTACACTGAAATATCTTTATGTCAGGCTTAATTTACTGCTATCTTGTTCAATAAAATGCCCCAAATTGGACTTGTTTCTGCCGTTAATTTGAGGCCAAGGTAATTCAGCTTATCTTTGGAGCTCGAGGTCTTCGTTGGGAACTGAAAGTCAAGATGTTTGCTTGCAAAGGAATTTGTTAATCACTAGTGAATTCGCGGCCGCCTGCAGGTCGACCATATGGGAGAGCTCCCAACGCGTTGGATGCATAGCTTGAGTATTCTATAGTGTCACCTAAATAGCTTGGCGTAATCATGGTCATAGCTGTTTCCTGTGTGAAATTGTTATCCGCTCACAATTCCACACAACATACGAGCCGGAAGCATAAAGTGTAAAGCCTGGGGTGCCTAATGAGTGAGCTAACTCACATTAATTGCGTTGCGCTCACTGCCCGCTTTCCAGTCGGGAAACCTGTCGTGCCAGCTGCATTAATGAATCGGCCAACGCGCGGGGAGAGGCGGTTTGCGTATTGGGCGCTCTTCCGCTTCCTCGCTCACTGACTCGCTGCGCTCGGTCGTTCGGCTGCGGCGAGCGGTATCAGCTCACTCAAAGGCGGTAATACGGTTATCCACAGAATCAGGGGATAACGCAGGAANGAACATGTGAGCAAAAGGCCAGCAAAAGGCCAGGAACCGTAAAAAGGCCGCGTTGCTGGCGTTTTTCNNNNGGNNNNGCCCCCNTGACANGATNNNAAAANTNNACGNNNAATTCANNNTGNNNAANCCNNCAGNNNNNAAANNNNCNNGGNTTNCCCNNGNAANCCCNNNNGGNNNCTCCTGNNNNNNNNNNNNNTNANNGAGAANNTNCCNNNNNNNNTTNGGNAGAGGGGNNTTNCNNNNNCCCCCNNTGGNNNNNNTNNTNNNNNNNNNNGNNNCCNANNNNNNNNTNGNNNNCNNNNNNNNNNNNNNNNNNNCNNNNNNNNNGNGNNNANNNCNNNCNANNNNNNNNNNNNNNNNNNNNNN

>763_T7_PREMIX Sample_Name=763_T7_PREMIX Chromat_id=6707508 Read_id=6646714 Version=1 Length=1330

NNNNNNANNNNNNGCTCCGGCCGCCATGGCGGCCGCGGGAATTCGATTACATAACAAATTCCTTTGCAAGCAAACATCTTGACTTTCAGTTCCCAACGAAGACCTCGAGCTCCAAAGATAAGCTGAATTACCTTGGACTCTCAAATTAACGGCAGAAACAAGTCCAATTTGGGGCATTTTATTGAACAAGATAGCAGTAAATTAAGCCTGACATAAAGATATTTCAGTGTAAGAAGATTGTCCCACTTGTGAACCTAAATACTATGCAAAATTCCTTATCTTTTTCAAATATTCTGGTTCTTAAGCATTTATTACACAAATTTATATGCAAAAGTAAAAAAGATTCTAGCTTTTACAGTACATATGAAACTTGTTCTTACACTATGAGAAAAATTAGCCTTCACCCACAAAATCAAGTTAAAATTCAAACGAGTAGAAAAACGAAAGCAAATAAACAAGATCCTAAACGGGAAAAAGAGAGCTTTTCTCAAGCAAAGCAATCACTAGTGAATTCGCGGCCGCCTGCAGGTCGACCATATGGGAGAGCTCCCAACGCGTTGGATGCATAGCTTGAGTATTCTATAGTGTCACCTAAATAGCTTGGCGTAATCATGGTCATAGCTGTTTCCTGTGTGAAATTGTTATCCGCTCACAATTCCACACAACATACGAGCCGGAAGCATAAAGTGTAAAGCCTGGGGTGCCTAATGAGTGAGCTAACTCACATTAATTGCGTTGCGCTCACTGCCCGCTTTCCAGTCGGGAAACCTGTCGTGCCAGCTGCATTAATGAATCGGCCAACGCGCGGGGAGAGGCGGTTTGCGTATTGGGCGCTCTTCCGCTTCCTCGCTCACTGACTCGCTGCGCTCGGTCGTTCGGCTGCGGCGAGCGGTATCAGCTCACTCAAAGGCGGTAATACGGTTATCCACAGAATCAGGGGATAACGCAGGAAAGAACATGTGAGCAAANGNCAGCAANGGCCAGGAACCGTAAAAAGGCNNNNNGCTGGGNTTTTTNNNNGGNCNNNCCCCNTGAAANNNNNNNAAANNNNNCCCCNAATNNNANGNNCCGAANCCCCNNGGGNNNAANNANNANNGCGTTNCCNNNNNNNNNCCCNNNGGNNNNNNNNNNNNNNNNNNNNNGNNANNNNNNNNNNNNNNCNNNNNCCTTNCNNNNNNNNGNNNNNNNNNNNNNNNNNNNNNNNNNNNNTNNNNNNNNNNNNNNNNCCCNNNNNNNNNNNNNNNNNNNNCCNNNCNNNNNCNNNNNNTTNNNNTTTTTTNCTCGCCAANNNTCNNNNNNNNNNNNNNNNNNNNGANNNNNNNNNNNNNGN

>3217_T7_PREMIX Sample_Name=3217_T7_PREMIX Chromat_id=6707480 Read_id=6646698 Version=1 Length=1254

NNNNNNNNNNNNNNNGCTNNNGCCGCCATGGCGGCCGCGGGAATTCGATTGCTTTGCTTGAGAAAAGCTCTCTCCTTTTCCCGTTTAGGATCTTGTTTATTTGCTTTCGTTTTTCTACTCGTTAAAATTTTAGCTTGATTTTGTGGGTGAATTATAACTTTACTCATAGTGCGAGAACAAGTTTCGTATGGACTGTAAAAGCTAGAATCTTTTTTACTTTTGCATATAAATTTGTGTAATAAATGCTTAAGAACCAGAATATTGAAAAAACAAAGGAATTCTACATAGTATTTAGGTTCACAAGTGGGACAATCTTCTTACAGTGAAATATCTTTATGTCAGGCTTAATTTACTGCTATTTTGTTCAGTAAAATGCCCCAAATTGGACTTGTTTCTGCCGTTAATTTGAGATCCAAGGTAATTCAGCTTATCTTTGGAGCTCGAGGTCTTCTTTGGGAACTGAAAGTCAAGATGGTCGCTTGCAAAGGAATTTGTTATGTAATCACTAGTGAATTCGCGGCCGCCTGCAGGTCGACCATATGGGAGAGCTCCCAACGCGTTGGATGCATAGCTTGAGTATTCTATAGTGTCACCTAAATAGCTTGGCGTAATCATGGTCATAGCTGTTTCCTGTGTGAAATTGTTATCCGCTCACAATTCCACACAACATACGAGCCGGAAGCATAAAGTGTAAAGCCTGGGGTGCCTAATGAGTGAGCTAACTCACATTAATTGCGTTGCGCTCACTGCCCGCTTTCCAGTCGGGAAACCTGTCGTGCCAGCTGCATTAATGAATCGGCCAACGCGCGGGGAGAGGCGGTTTGCGTATTGGGCGCTCTTCCGCTTCCTCGCTCACTGACTCGCTGCGCTCGGTCGTTCGGCTGCGGCGAGCGGTATCAGCTCACTCAAAGGCGGTAATACGGTTATCCACAGAATCAGGGGATAACGCAGGAANGAACATGTGAGCAAAAGGCCAGCAAAAGGCCAGGAACCGTAAAAAGGCCGCGTTGCTGGCGTTTTTCCATAGGCTCCNCCCCCNTGACANNNTNNNNAAANTCGACGCTCAAGTCAAANTGNNNAANCCCGACGGNCTATAANNNNCCNGGNNTTNCCCNNGNAANCNNNNNNNNNNNNCTCCGGTTCCAANNTGNNNTTACNNNNNNNNNTCCCNTTTNNNNNNGGNNNNNGGGNNTNNNNAANNNNACNGAGNNANNNCCNNNGGNNNNNNNNNCCNANNGGGNNTGNNNAAAAANN

>4335_T7_PREMIX Sample_Name=4335_T7_PREMIX Chromat_id=6707490 Read_id=6646681 Version=1 Length=1270

NNNNNNNNNNNNNCNGCTNNNGCCGCCATGGCGGCCGCGGGAATTCGATTGCTTTGCTTGAGAAAAGCTCTCTCCTTTTCCCGTTTAGGATCTTGTTTATTTGCTTTCGTTTTTCTACTCGTTAAAATTTTAACTTGATTTTGTGGGTGAATTATAACTTTACTCATAGTGCGAGAACAAGTTTCGTATGGACCGTAAAAGCTAGAATCTTTTTTACTTTTGCATATAAATTTGTGTAATAAATGCTTAAGAACCAGAATATTGAAAAAACAAAGGAATTCTACATAGTATTTAGGTTCACAAGTGGGACAATCTTCTTACAGTGAAATATCTTTATGTCAGGCTTAATTTACTGCTATTTTGTTCAGTAAAATGCCCCAAATTGGACTTGTTTCTGCCGTTAATTTTCCAAGGTAATTCAGCTTATCTTTGGAGCTCGAGGTCTTCGTTGGGAACTGAAAGTCAAGATGTTTGCTTGCAAAGGAATTTGTTATGTAATCACTAGTGAATTCGCGGCCGCCTGCAGGTCGACCATATGGGAGAGCTCCCAACGCGTTGGATGCATAGCTTGAGTATTCTATAGTGTCACCTAAATAGCTTGGCGTAATCATGGTCATAGCTGTTTCCTGTGTGAAATTGTTATCCGCTCACAATTCCACACAACATACGAGCCGGAAGCATAAAGTGTAAAGCCTGGGGTGCCTAATGAGTGAGCTAACTCACATTAATTGCGTTGCGCTCACTGCCCGCTTTCCAGTCGGGAAACCTGTCGTGCCAGCTGCATTAATGAATCGGCCAACGCGCGGGGAGAGGCGGTTTGCGTATTGGGCGCTCTTCCGCTTCCTCGCTCACTGACTCGCTGCGCTCGGTCGTTCGGCTGCGGCGAGCGGTATCAGCTCACTCAAAGGCGGTAATACGGTTATCCACAGAATCAGGGGATAACGCAGGAANGAACATGTGAGCAAAAGGCCAGCAAAAGGCCAGGAACCGTAAAAAGGCCGCGTTGCTGGCGTTTTTCCATAGGCTCCGCCCCCNTGACAANNNTCACAAAANTCGACGCTCAAGTCAAAGGTGGNAAACCNGNNNGGNNATAAAANNNCNNNNCGTTNCCCNNGNAANNNNNNNNNNNNNCTCCGGTTCCAACCTGNNNTNAANNGAAANNNNNNCCCNTTTNNNNNNCTGNNNGNNGGNNNNTTTTNNNNNNNNCCNNNAGNNNNNNNANNNNNNNNNTGNNNNNNNCCTNNNNNNNNNNNNNNNNCNNNNNNNNNNN
